# New Types and Dosages for the Manufacture of Low-Energy Cements from Raw Materials and Industrial Waste under the Principles of the Circular Economy and Low-Carbon Economy

**DOI:** 10.3390/ma16020802

**Published:** 2023-01-13

**Authors:** Sergio Martínez-Martínez, Luis Pérez-Villarejo, Dolores Eliche-Quesada, Pedro J. Sánchez-Soto

**Affiliations:** 1 Department of Chemical, Environmental and Materials Engineering, University of Jaén, Campus Las Lagunillas s/n, 23071 Jaén, Spain; 2 Institute of Materials Science of Sevilla (ICMS), Joint Center of the Spanish National Research Council (CSIC), University of Sevilla, 41092 Sevilla, Spain

**Keywords:** low-energy cements, waste products, belite, clinker, circular economy, construction materials, CO_2_ emissions, low-carbon economy

## Abstract

The cement manufacturing industry is one of the main greenhouse gas emission producers and also consumes a large quantity of raw materials. It is essential to reduce these emissions in order to comply with the Paris Agreement and the principles of the circular economy. The objective of this research was to develop different types of cement clinker blends using industrial waste and innovative design to produce low-energy cement. Several types of waste have been studied as alternative raw materials. Their main characteristics have been analyzed via X-ray fluorescence (XRF), X-ray diffraction (XRD), Attenuated total reflectance Fourier trans-form infrared spectroscopy (ATR-FTIR), thermal analysis (TG-DTG-DSC) and scanning electron microscopy and energy dispersive X-ray spectroscopy analysis (SEM-EDS). The results obtained from the experimental work carried out in this research focused on the study of crude blends for low-energy cement created from industrial waste. The effect of the addition of different industrial waste types, as a substitution for raw materials, in the production of low-energy cement with high dicalcium silicate content has been investigated. Thus, the dosage design has been performed using modified Bogue equations and quality indexes (LSF, AM, and SM). The calculations of both the modified Bogue equations and quality indexes necessitate knowledge of the weight percentages of CaO, SiO_2_, Al_2_O_3_, and Fe_2_O_3_, determined via XRF. In this theoretical design of the different blends, it has been established that a dicalcium silicate ratio of 60–65 wt % and an LSF of 78–83% as the limit are values common to all of them. The calculation basis for the crude blends has been based on calcined materials. Therefore, the chemical composition was established, following this premise. Thus, it was possible to develop cement clinker blends with compositions of 50 wt % and 100 wt % using industrial wastes. This research has shown that the clinkerization process is one of the main options for the valorization of waste and its consideration for inclusion as a raw material within the circularity of the cement industry’s production process. Thus, waste is used as a raw material for the production of a more useful substance, taking into account the fundamental principles of the circular economy.

## 1. Introduction

Humanity is at a critical juncture in terms of successfully tackling the greatest challenge of our time, climate change. Since the outbreak of the Industrial Revolution, and most markedly in the last century and a half, humankind has been responsible for a dramatic increase in greenhouse gas (GHG) emissions (Appendix A, Table A1), produced largely by industrialization and the burning of fossil fuels, as well as by deforestation and large-scale agriculture. Among the greenhouse gases, the most abundant is carbon dioxide (CO_2_), accounting for about two-thirds of all greenhouse gases [1]. This GHG is mainly produced by burning fossil fuels.

In 2015, after four years of negotiations, the Paris Agreement was adopted at the twenty-first session of the Conference of the Parties to the United Nations Framework Convention on Climate Change (COP21) and the eleventh session of the Conference of the Parties, serving as the meeting of the Parties to the Kyoto Protocol (COP-MOP11) in Paris. This global, legally binding climate change agreement obliges all countries to participate in global GHG reductions [2].

Taking the above into account, in December 2019, the “European Green Deal” was launched within the European Union [3]. This European Green Pact sets out an action plan to boost resource efficiency by moving to a clean and circular economy, as well as restoring biodiversity and reducing pollution.

In addition to the increase in GHG emissions, the expansion of industrial activity also increases the amount of waste generated. Currently, most of the waste generated in the construction or mining industry is disposed of in landfills, which, in turn, generates numerous environmental problems and higher costs for companies. Therefore, it is essential to find new methods to absorb the waste generated by the production process.

One author describes the concept of the circular economy, as follows: “A circular economy aims to maintain the value of products, materials, and resources for as long as possible by returning them to the product cycle at the end of their use, while minimizing the generation of waste. The fewer products we discard, the less material we extract, the better for our environment. This process starts at the very beginning of a product’s lifecycle: smart product design and production processes can help save resources, avoid inefficient waste management, and create new business opportunities” [4]. In 2015, the European Commission adopted the ambitious EU Action Plan for the Circular Economy, which sets out a program of 54 concrete actions, covering the entire product life cycle from production and consumption to waste management, as well as the secondary raw materials market [4].

Cement is one of the main building materials that is manufactured and used worldwide, with a global production rate in 2021 of 4270 Mt. China and India are the world’s largest producers, accounting for approximately 55% and 8% of the world’s total cement production, respectively [5]. Good durability performance and compatibility with other construction elements allow the production of composite materials with improved properties [6]. Conversely, the cement manufacturing industry is one of the main industries in terms of GHG emissions, taking as a reference the fact that approximately 0.59 tonnes of CO_2_ are generated for each tonne of cement produced, according to data from the year 2021 [5]. The total CO_2_ emissions produced by the cement industry account for approximately 8% of global CO_2_ emissions [7]. These CO_2_ emissions are released in two main ways: 60% of emissions is due to the thermal decomposition of CaCO_3_ into CaO and CO_2_, while 40% is due to the consumption of thermal energy required for the production process [8,9,10,11]. Depending on the raw materials and the production process, 60 to 130 kg of fuel oil, or its equivalent, is required for each tonne of cement produced, as well as about 110 kWh of electricity [12].

In addition to the above, the cement manufacturing industry consumes a large number of raw materials that are rich in calcium oxides, silica, alumina, and iron oxide, which can be of mineral origin, mainly derived from clayey, limestone, or loamy materials, or from waste products [13]. The extraction and processing of these raw materials for the cement manufacturing industry are also associated with numerous costs and GHG emissions, as well as the corresponding deterioration of the environment through the generation of degraded areas.

Considering all this, it is essential to reduce GHG emissions from the cement manufacturing industry, in order to comply with the Paris Agreement, as well as to use it as a lever for change in the new concept of the circular economy, bearing in mind that the cement industry possesses a great capacity to absorb industrial wastes that could be used as raw material in the cement manufacturing process.

Clinker is the main ingredient in cement, with the CO_2_ emissions generated in cement manufacture being directly proportional to the amount of clinker used. Between 2015 and 2020, the clinker–cement ratio was estimated to increase by approximately 1.6%/year, on average, reaching 0.72 in 2020 [5]. Thus, direct CO_2_ emissions from cement production increased from 2203.0 Mt CO_2_ in 2015 to 2526.8 Mt CO_2_ in 2021 [5]. Ordinary Portland cement (OPC) clinker is generally composed, on average, of 67% CaO, 22% SiO_2_, 5% Al_2_O_3_, and 3% Fe_2_O_3_, in addition to ~3% of other minor oxides. Depending on the temperature of the process and the proportion of the oxides, a series of solid-state reactions take place that give rise to the formation of the phases that make up the cement clinker [14,15]. In OPC, the proportions of each of these phases range from 50–70 wt % of C_3_S (alite or tricalcium silicate), 15–30 wt % of C_2_S (belite or dicalcium silicate), 5–10 wt % of C_3_A (tricalcium aluminate) and 5–15 wt % of C_4_AF (tetracalcium alumino-ferrite), with the characteristics and properties of the cement varying according to the variability of its composition [14,15].

Belitic Portland cement (BPC) or belitic cement, compared to OPC, has a higher proportion of C_2_S compared to C_3_S in their composition. In addition, C_2_S has lower energy requirements in its formation process. This inversion of the proportions of the predominant silicate ratio in the BPC clinker leads to a reduction in CO_2_ emissions, due to two main factors: the lower clinkering temperature requirement, which is approximately 100–150 °C lower than OPC [16], implying a reduction in energy consumption and a reduction in the need for limestone as a raw material. This, in turn, reduces the CO_2_ emissions due to the decarbonation reaction during the clinkerization process in the kiln. The reduction in the energy demand of BPCs compared to OPCs can be accounted for in savings of approximately 150 kJ/kg [17]. In addition to this, the energy factor accounts for 30–40% of the total cost of production [18].

**Figure 1:**
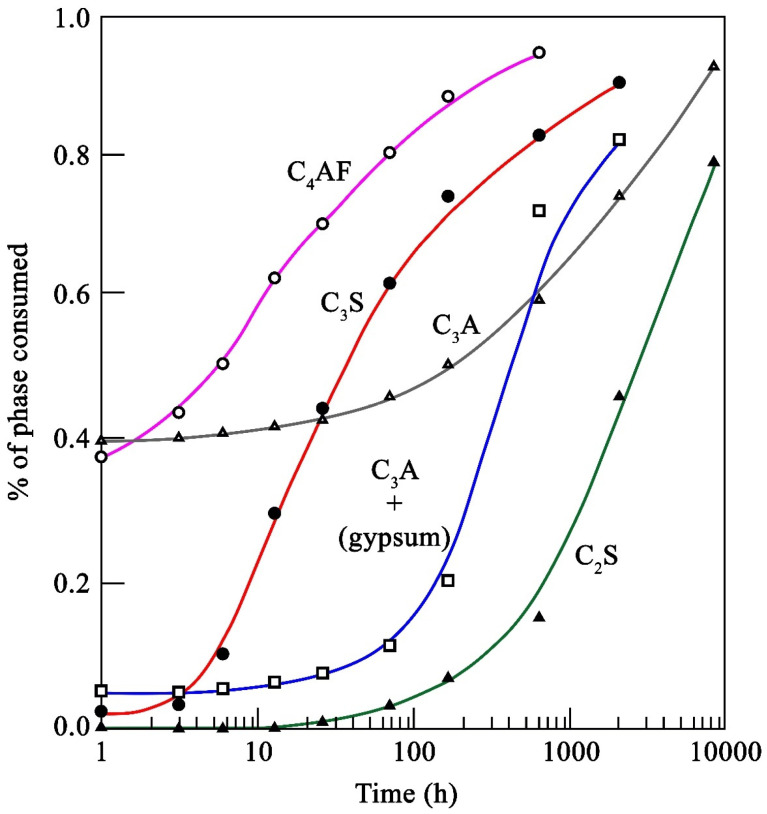
Typical hydration kinetics of the pure phases of cement clinker at room temperature (adapted from reference [19]).

In contrast, cements rich in C_2_S have lower initial strengths than OPC, mainly because belite has slower hydration kinetics than alite (Figure 1). However, the hydration kinetics in belite will depend on the polymorph obtained during the manufacturing process, which in belite can be of five different forms, known as α, α’_H_, α’_L_, β, and γ.

Thus, the objective of this research is the development of different types of cement clinker blends with a high belite content, the main characteristic of which is the use of industrial wastes in their composition as raw materials, as well as their design for the production of low-energy cement clinkers, with a reduction in the manufacturing temperature of OPCs of approximately 100–150 °C, thereby improving the circularity of cement production and CO_2_ emissions. The motivation for this research is the possibility of manufacturing cement clinker from industrial wastes, either at a high percentage of 50 wt % or the total substitution of raw materials, i.e., a cement clinker composed entirely of industrial waste.

## 2. Materials and Techniques

### 2.1. Samples and Sample Preparation

**Figure 2:**
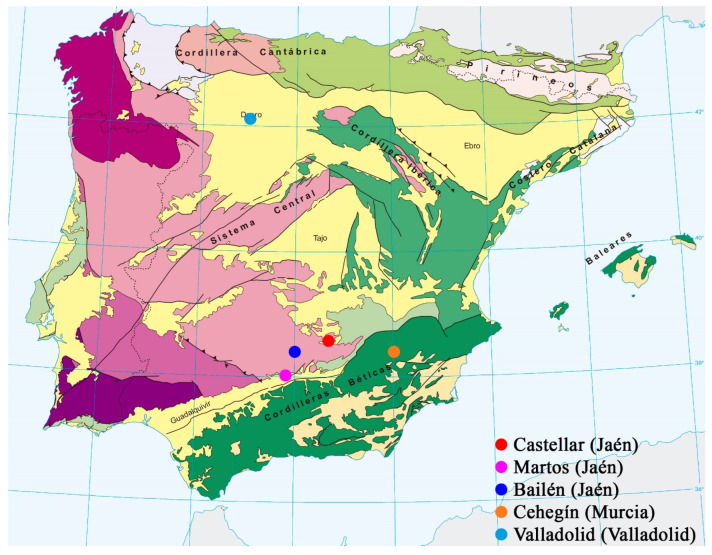
Map of the Iberian Peninsula and the Balearic Islands, showing the main geological units and the location of the points of origin of Spain’s raw materials and industrial waste [20].

A total of eight raw materials and three industrial waste types have been selected, which come from different geographical areas (Figure 2). As indicated in the Introduction, the main materials to be used for clinker manufacture with a high dicalcium silicate (belite, C_2_S) content are rich in calcium oxide (CaO) and silicon dioxide (SiO_2_), in terms of their chemical composition. For clinker design, the necessary limit to the composition of aluminum oxide (Al_2_O_3_), ferric oxide (Fe_2_O_3_) and magnesium oxide (MgO) must be considered. This composition will directly influence the clinker quality indices, calculated by means of modified Bogue equations, as well as the expansivity of the cement.

In this way, a series of raw materials and industrial wastes have been initially selected, considering both their initial chemical composition and their production capacity. In all the cases, materials are incorporated that, due to their versatility and widespread use, can be found on all five continents. Thus, the main source of CaO will be limestone and marble-cutting sludge. The main source of SiO_2_ will be selected clays (yellow, black, and red clays) and the crushed waste material, chamotte. Salt slags from secondary aluminum production (PAVAL) will be used as the Al_2_O_3_ content corrector. Finally, gypsum will be used as a setting regulator for the cement paste.

ANAYA limestone (designated as C_A): This limestone comes from Castellar (Jaén, Spain) (Figure 2). From the geological point of view, the area of exploitation is located in the outer reaches of two large geological units: the Iberian Massif and the Baetic Mountain Range, this also being the northeastern apex of the Guadalquivir basin [21]. The sample was supplied by the trading company, ÁRIDOS ANAYA, S.L. The extraction works are carried out in open-cast mines by blasting benches of approximately 15–17 m in height using explosives.

SANTO NICASIO limestones 1 and 2 (designated as C_SN_1 and C_SN_2, respectively): The samples come from Martos (Jaén, Spain) (Figure 2). From the geological point of view, the exploitation area is located in the northern sector of the Baetic mountain range, at the boundary of the Outer Zones with the Guadalquivir Depression [22]. The limestone was supplied by the trading company, ÁRIDOS SANTO NICASIO, S.L. The extraction works are carried out in open-cast mines by blasting benches of approximately 20 m high with explosives.

TITAN limestone (designated C_T): This limestone comes from Patras (Greece). The sample was supplied by the trading company TITAN GREECE. This limestone is extracted in open-cast mines by blasting with explosives.

The three clays used in this research, called yellow clay, black clay, and red clay (designated as A_A, A_N and A_R respectively), come from Bailén (Jaén, Spain) (Figure 2). From the geological point of view, the area where these clays are sourced is situated on the territory where two of the main morphological units of the main range where the Iberian Peninsula can be divided: the Iberian Plateau and the Guadalquivir Depression [23]. The clays were supplied by the trading company, ARCILLAS BAILÉN, S.L. The extraction work is carried out via open-cast mines using mechanical means (a backhoe or bulldozer).

The industrial wastes or by-products used in this research are:

- Chamotte (designated as CHM), obtained by grinding or crushing fragments of fired ceramic materials (produced at high temperature) that are defective, either because the kiln has not reached the necessary temperature in the sintering process, because the material has spent less time than necessary in the kiln for sintering, or because of the appearance of cracks, defects, etc., during the manufacturing process. In this case, the defective ceramic pieces were supplied by the trading company LADRILLOS BAILÉN S.A., located in Bailén (Jaén, Spain) (Figure 2).
- Cutting and polishing marble sludges (designated as MRM) sourced from Cehegín (Murcia, Spain) (Figure 2). From a geological point of view, the study area is mainly located within the Subbetic zone [24]. In terms of the extraction of the marble blocks from which the sludge was sourced, the main process used was the cutting of marble blocks with a wire cutter (diamond wire), which generates sludge with a grain size of less than 0.15 mm [25]. Likewise, sludge from marble block-cutting processes using looms or block cutters, as well as from the marble slab-polishing process, have also been used.
- Secondary aluminum production salt slag (designated as PVL), also known as PAVAL, comes from the factory owned by the trading company, BEFESA MEDIO AMBIENTE, S.L., located in Valladolid (Spain) (Figure 2). This sample has an aluminum oxide (Al_2_O_3_) content of around 70%, so it will be used as a corrector of this oxide in clinker obtained from industrial waste.

All the materials, except the gypsum (designated as Y), were received in the laboratory and then subjected to a moisture control process. For the calculation of the moisture percentage, carried out in triplicate, Equation (1) is used, where part of the initial material sample (m_i_) is weighed with an analytical balance and placed in a laboratory oven at 105 °C for 24 h. After 24 h, the material (m_f_) is weighed again, repeating this process until a constant value is reached between two weighings. The moisture percentage is determined according to the formula:

% moisture = ((m_i_ − m_f_)/m_i_) × 100 (1)

The moisture contents can be seen in Table 1, although these values will depend to a large extent on the period of year when the materials are received, interfering with the humidity of the environment and the humidity caused by rainfall in the stockpile areas, especially in mining operations.

Once the moisture content of the raw materials has been determined, the same drying process is carried out with the entire volume of material received, in order to facilitate the subsequent grinding and sieving of all the samples. It is very important to obtain a homogeneous particle size that allows all the reactions in the clinkerization process to be carried out correctly, as the control of the particle size influences the physical and mechanical characteristics of the final product [26]. In this investigation, all materials were sieved to a particle size of 100 μm or less.

### 2.2. Techniques

The experimental techniques used for the characterization of the raw materials and industrial wastes included establishing their chemical composition, determined by X-ray fluorescence (XRF, PANalytical^®^ model Zetium, Malvern, UK), mineralogical composition, determined by X-ray diffraction (XRD, PANalytical^®^ model Empyrean, Malvern, UK), thermogravimetric analysis, derivative thermogravimetric analysis, and differential scanning calorimetry (TG-DTG-DSC, NETZSCH^®^ model STA 449 F3 Jupiter, Selb, Germany), a morphological study via scanning electron microscopy and chemical analysis by energy-dispersive X-ray spectroscopy analysis (SEM, Carl Zeiss^®^ model MERLIN, Jena, Germany, EDS, Oxford Inca Energy 350X-MAX 50, Abingdon, UK), as well as functional group identification by attenuated total reflectance Fourier transform infrared spectroscopy (ATR-FTIR, Bruker^®^ model Tensor 27, Billerica, MA, USA).

### 2.3. Dosage Design, Modified Bogue Equations and Quality Indexes

As discussed above, the main difference in the design of ordinary Portland cement (OPC) and cement with a high content of C_2_S is the proportion of tricalcium silicate (C_3_S) and dicalcium silicate (C_2_S) in the cement clinker. This, in turn, is associated with the ratio of one of the most important quality indices, the lime saturation factor (LSF). The LSF marks the ratio between the lime contained in the clinker and the maximum amount of lime that can be set by the components at their maximum basicity; in the case of values lower than 100, they have a composition that can withstand subsequent lime additions. The opposite scenario with values higher than 100 indicates a composition with a theoretical excess of lime [27].

In addition to the LSF, there are two other quality indices to be taken into account in the clinker design. These two quality indices are the aluminum modulus (AM) and the silica modulus (SM). The AM establishes a ratio between the Al_2_O_3_ and Fe_2_O_3_ contents, which is equivalent to relating the “fluxing” components, i.e., C_3_A and C_4_AF [27]. The SM characterizes the ratio between the solid phase and the liquid phase; its value indicates the specific weight of the silicates in the clinker, compared to the fluxes. The higher the SM, the lower the percentage of liquid phase, and the worse the characteristics of the raw blend during the firing process of lime production [27].

For the design of the raw materials, in addition to theoretically calculating the quality indices that the cement clinker will have, the theoretical proportions of the mineralogical phases of the manufactured clinker are calculated, which are C_3_S, C_2_S, C_3_A, and C_4_AF. The approximate calculation of these theoretical phases of the cement clinker is performed using modified Bogue equations (Equations (2)–(5)) [6,28,29]. It is important to note that for the calculation of the crude blends and, therefore, the calculation of the addition of the different industrial wastes, the proportions of the main oxides (CaO, SiO_2_, Al_2_O_3_, and Fe_2_O_3_) must be kept constant.

C_3_S = 4.0710·CaO − 7.6024·SiO_2_ − 1.4297·Fe_2_O_3_ − 6.7187·Al_2_O_3_ (2)

C_2_S = 8.6024·SiO_2_ + 1.0785·Fe_2_O_3_ + 5.0683·Al_2_O_3_ − 3.0710·CaO (3)

C_3_A = 2.6504·Al_2_O_3_ − 1.6920·Fe_2_O_3_ (4)

C_4_AF = 3.0432·Fe_2_O_3_ (5)

The equations used for the calculation of LSF (Equation (6)), AM (Equation (7)), and SM (Equation (8)) are as follows [6,27,30]:

LSF = (100·CaO)/(2.8·SiO_2_ + 1.18·Al_2_O_3_ + 0.65·Fe_2_O_3_) (6)

AM = Al_2_O_3_/Fe_2_O_3_ (7)

SM = SiO_2_/(Al_2_O_3_ + Fe_2_O_3_). (8)

The calculations of both modified Bogue equations and the quality index imply the percentages by weight of each oxide (CaO, SiO_2_, Al_2_O_3_, and Fe_2_O_3_).

A Microsoft Excel^®^ spreadsheet designed by researchers at the METLAB laboratory of the Department of Chemical Engineering of the University of Patras (Patras, Greece) was used for the design of the crude blends [6]. For the purposes of this theoretical design, a C_2_S ratio of 60–65% and an LSF of 78–83% are used as limiting values that are common to all of them [31,32].

## 3. Results and Discussion

### 3.1. Characterization of Raw Materials and Industrial Wastes

#### 3.1.1. Chemical Results

The results of the chemical composition of the raw materials and industrial wastes, expressed in oxides, as determined by the X-ray fluorescence technique (XRF), as well as the loss on ignition (LOI) or calcination at 1000 °C, are presented in Table 2 and Table 3.

The limestones (Table 2) are mainly composed of CaO, with values varying between 34.54 wt % by weight of the C_A and 46.54 wt % of the C_T, noting that between the two fractions obtained from the SANTO NICASIO limestone (C_SN_1 and C_SN_2), there are no significant differences (41.86 wt % and 42.77 wt %). The main difference between the limestones is fundamental and is found in their magnesium oxide content, which is a determining factor in the calculation of the raw material dosages. This will, to a great extent, be responsible for the future expansivity of the cement. In this sense, it is possible to observe the great variation between C_A (19.12 wt %), C_SN_1 (4.59 wt %), C_SN_2 (4.63 wt %), and C_T (0.63 wt %). It should be borne in mind that the calculation basis for the crude blends is based on calcined materials, i.e., eliminating the corresponding losses on ignition (LOI), so that the percentage of magnesium oxide, as is the case with the other chemical elements, will be higher. Furthermore, C_A has higher ignition losses than the rest (46.31 wt %), the lowest being those of C_T (38.45 wt %). In the case of C_T, the lower content of magnesium oxide and the loss on ignition is compensated for by a higher percentage of silica (10.51 wt %).

The clays (Table 2) are mainly composed of SiO_2_, with a content of more than 44 wt % in all of them. A_A and A_N have very similar percentages of Al_2_O_3_ (~10 wt %), CaO (~17 wt %), Fe_2_O_3_ (~5 wt %), K_2_O (~2 wt %), and MgO (~1.8 wt %). However, A_N has a higher percentage of SO_3_ (8.26 wt %), it being practically nil in A_A. A_R has a higher content of Al_2_O_3_ (19.81 wt %), Fe_2_O_3_ (7.71 wt %), K_2_O (5.47 wt %), and MgO (3.43 wt %) than the other clays, although its CaO content is lower (3.89 wt %) and its SO_3_ content is almost zero (0.05 wt %). The ignition losses are generally associated with the physically retained water and structural OH of silicates, as well as the decomposition process of the carbonates. The values obtained are higher for the A_A sample (12.23 wt %) compared with the lower values for the A_R (9.07 wt %).

Gypsum (Y) (Table 3), which will be used as a setting regulator in the hydrated cement pastes, is mainly composed of SO_3_ (38.53 wt %) and CaO (24.25 wt %). The sample contains smaller amounts of SiO_2_ and MgO, as well as ignition losses of less than 23 wt %.

The CHM waste (Table 3) is mainly characterized by its high SiO_2_ content (51.23 wt %), with significant additional contents of CaO and Al_2_O_3_ that are always above 10 wt %, as well as Fe_2_O_3_ (6.92 wt %). It can be seen, in general terms, that chamotte waste has a chemical composition similar to that of the clays.

The MRM waste (Table 3) is basically composed of CaO (52.14 wt %), although it presents very high ignition losses (43.31 wt %), due, to a great extent, to the decomposition of the calcium carbonates in the form of CO_2_ as evolved gas. They also contain very low quantities of impurities in the form of Fe_2_O_3_, MgO, or SO_3_, always in percentages of less than 0.20 wt %.

The PVL waste (Table 3) is mainly composed of aluminum-rich oxide and hydroxide phases [33,34]. Their Al_2_O_3_ composition value is high (65.40 wt %), a finding that coincides with the data provided by the producer [35]. The MgO (8.28 wt %) and SiO_2_ (5.07 wt %), as well as the Na_2_O and F (~1 wt %) contents, are also noteworthy, albeit to a lesser extent. As already mentioned in the case of limestones, the MgO value must be taken into account when calculating the different raw material dosages because of its effects on the future expansivity of the cement.

#### 3.1.2. Mineralogical Composition

**Figure 3:**
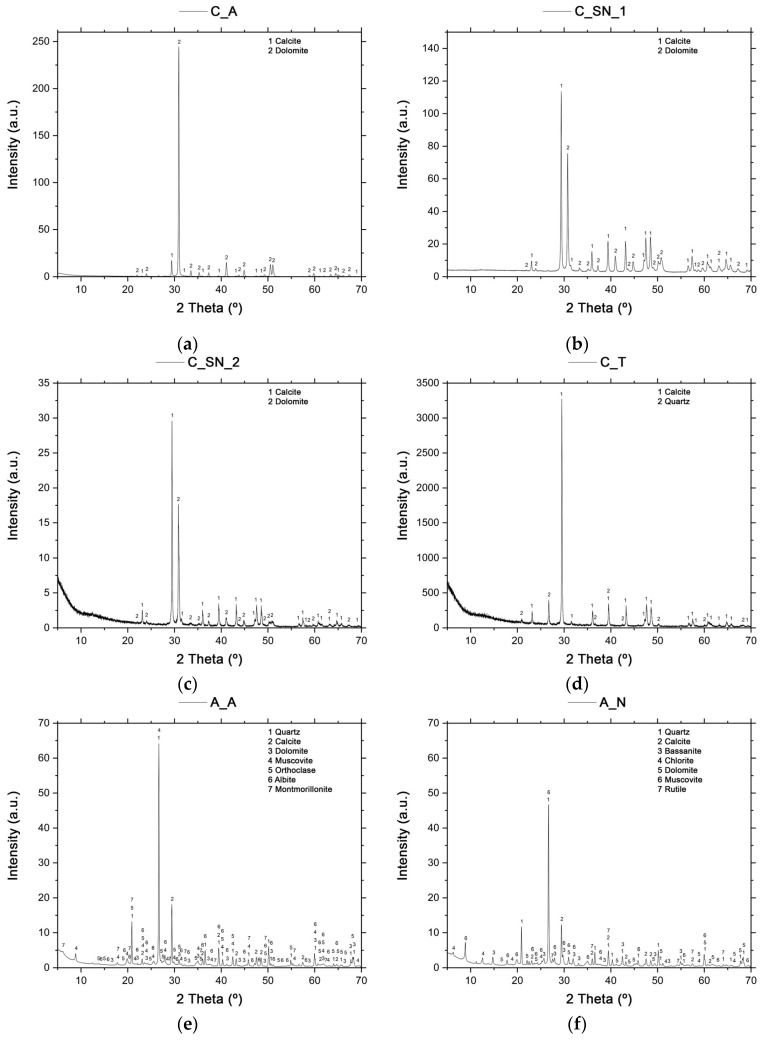

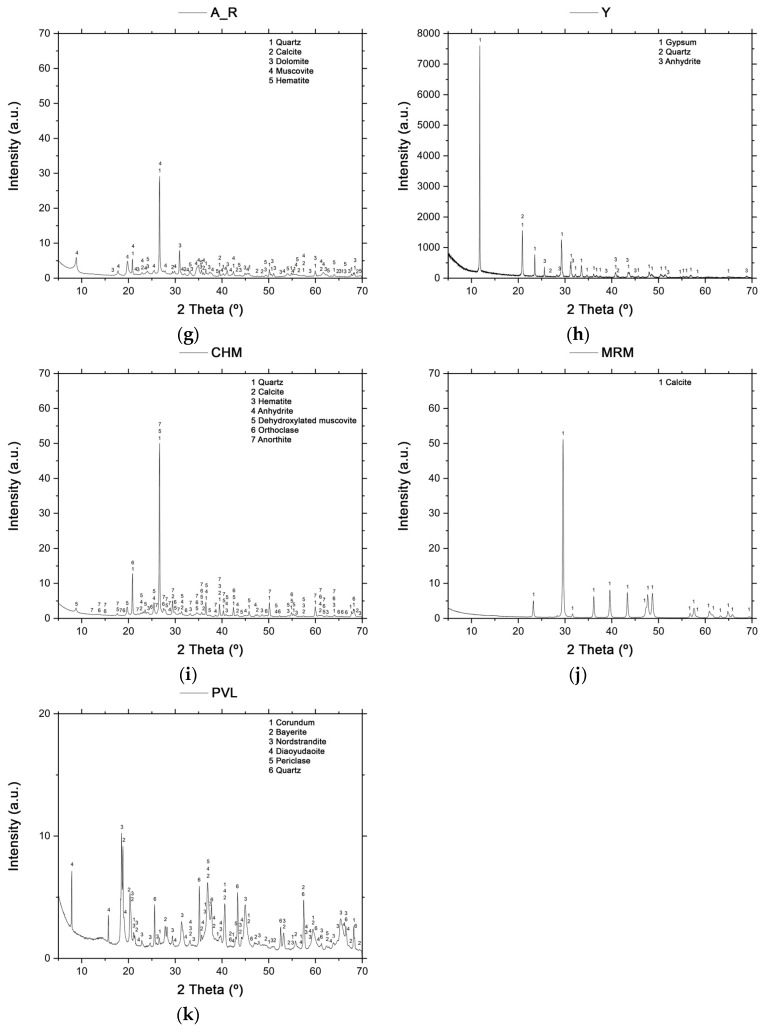
X-ray diffractograms of: (**a**) C_A; (**b**) C_SN_1; (**c**) C_SN_2; (**d**) C_T; (**e**) A_A; (**f**) A_N; (**g**) A_R; (**h**) Y; (**i**) CHM; (**j**) MRM; (**k**) PVL.

The crystalline phases identified for sample C_A (Figure 3a) are calcite (CaCO_3_) and dolomite [CaMg(CO_3_)_2_]. More intense diffraction peaks are observed for dolomite, which is in agreement with the high percentage of magnesium oxide determined in its chemical composition (Table 2). This limestone can therefore be considered a dolomitic limestone. The crystalline phases identified for samples C_SN_1 (Figure 3b) and C_SN_2 (Figure 3c) are calcite and dolomite. It is clear that the two limestones have a similar XRD pattern, according to their chemical composition results. Compared to the results obtained for C_A, a lower intensity of the diffraction peak corresponding to dolomite is observed in the two diffractograms of C_SN_1 and C_SN_2. This is due to the lower amounts of magnesium oxide in their chemical composition (Table 2). The crystalline phases identified for C_T (Figure 3d) are calcite and quartz (SiO_2_). These results agree with the chemical composition of the limestone (Table 2), with CaO accounting for 46.54 wt % and SiO_2_ for 10.51 wt %. Unlike the diffractograms of C_A, C_SN_1, and C_SN_2, dolomite is not identified in C_T. This is due to the almost scarce presence of MgO in the limestone composition (Table 2); therefore, the conditions for the substitution of calcium ions by magnesium in calcite do not occur.

The crystalline phases identified for sample A_A (Figure 3e) are quartz, calcite and dolomite, muscovite (illite mica) [KAl_2_(AlSi_3_O_10_)(OH)_2_], and feldspars such as orthoclase (KAlSi_3_O_8_) and albite (NaAlSi_3_O_8_), as well as montmorillonite (magnesium aluminum silicate: MgOAl_2_O_35_SiO_2_xH_2_O). In the case of sample A_N (Figure 3f), the crystalline phases that were identified are quartz, calcite and dolomite, bassanite or gypsum hemihydrate (CaSO_4_.1/2H_2_O), chlorite [(Al,Fe,Mg)_4–6_(Al,Si,Fe)_4_O_10_(OH,O)_8_], muscovite (illite mica) and rutile (TiO_2_). For sample A_R (Figure 3g), the crystalline phases identified are quartz, calcite, and dolomite, muscovite (illite mica), and hematite (Fe_2_O_3_), the latter crystalline phase being responsible for the red color of the clay.

The crystalline phases identified for the sample of gypsum (Y) (Figure 3h), the setting regulator, are calcium sulfate dihydrate or pure phase mineral gypsum (CaSO_4_–2H_2_O), quartz, and anhydrite (CaSO_4_). Quartz has been identified due to the high percentage of SiO_2_ in the chemical analysis (Table 3), this not being associated with silicates in the sample.

The crystalline phases identified for the CHM waste (Figure 3i) are quartz, calcite, hematite, anhydrite, dehydroxylated muscovite (illite mica), feldspars such as orthoclase and calcium, and an aluminum feldspar called anorthite (CaAl_2_Si_2_O_8_), which is a product of the heat treatment [6].

In the MRM diffractogram (Figure 3j), only calcite can be identified, which fits perfectly with the standard CaCO_3_ pattern [6].

The crystalline phases identified for the PVL waste (Figure 3k) are corundum (α-Al_2_O_3_), which is a mineral belonging to the hematite group, bayerite [γ-Al(OH)_3_], which is an aluminum hydroxide crystallizing in the monoclinic system, nordstrandite [Al(OH)_3_], which is an aluminum hydroxide crystallizing in the triclinic system, diaoyudaoite (NaAl_11_O_17_), which is an aluminum oxide containing sodium in solid solution, periclase (MgO), and quartz. According to the characteristics provided by the producer and reported in [35], PVL also has fluorite (CaF_2_) in its mineralogical composition, in a percentage of 1–2%, as well as halite (NaCl), in a percentage of 0–1%, both mineralogical phases not being identified by XRD. A large number of aluminum-based mineral phases coincide with the origin of the waste obtained in the recycled aluminum process. In addition, the presence of compounds such as fluorite is due to their use as scorification salts during the recycling process.

#### 3.1.3. Thermal Analysis (TG-DTG-DSC)

**Figure 4:**
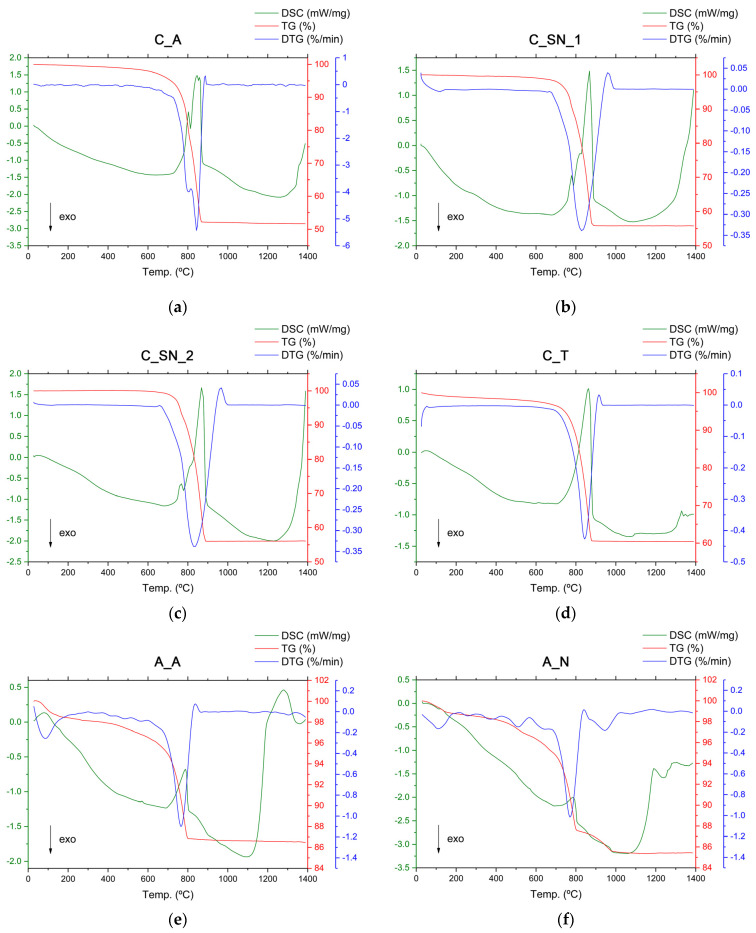

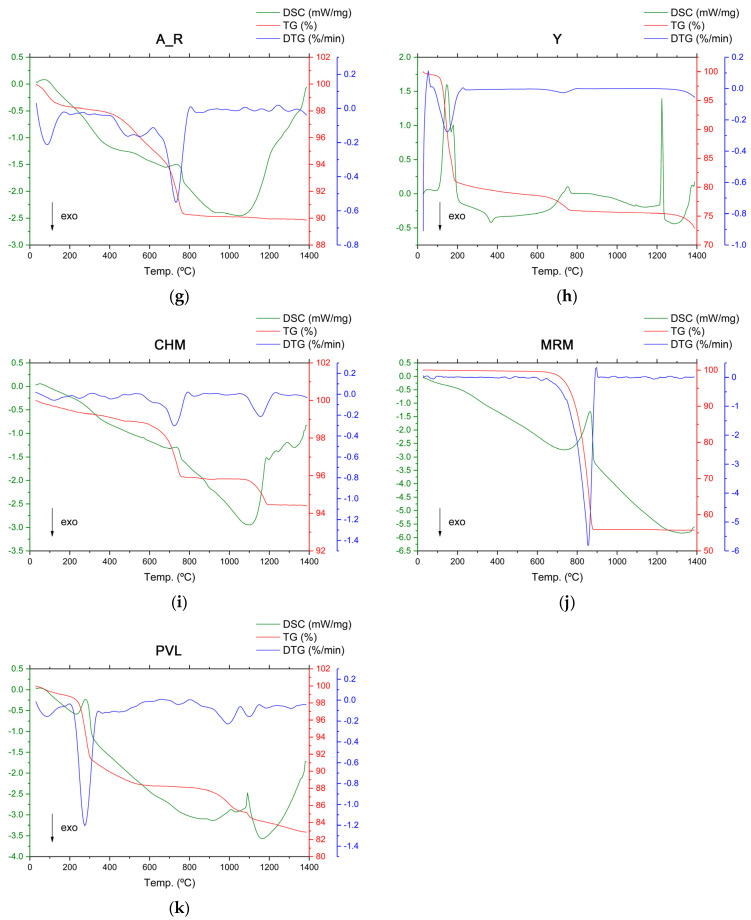
TG (red line), DTG (blue line), and DSC (green line) analysis of: (**a**) C_A; (**b**) C_SN_1; (**c**) C_SN_2; (**d**) C_T; (**e**) A_A; (**f**) A_N; (**g**) A_R; (**h**) Y; (**i**) CHM; (**j**) MRM; (**k**) PVL.

The analyses were carried out on C_A (Figure 4a), C_SN_1 (Figure 4b), and C_SN_2 (Figure 4c), which are mineralogically composed of calcite and dolomite, according to the XRD patterns. In the thermogravimetric (TG) curve, the calcite and dolomite show a total weight loss at up to 880 °C of 48 wt % and 44 wt %, respectively, with the limestones C_SN_1 and C_SN_2 showing equivalent weight losses. This weight loss is due to the decomposition of the calcium and magnesium carbonates in two consecutive stages. The analyses carried out on C_T (Figure 4d), which is mineralogically composed of calcite and quartz, as indicated by XRD data, show a total weight loss of 39 wt % up to 880 °C in the TG curve, which is due to the decomposition of calcium carbonate (Equation (9)).

According to the differential scanning calorimetry (DSC) analysis of limestones C_A, C_SN_1, and C_SN_2, the first endothermic reaction is observed at 803 °C, 780 °C, and 775 °C, respectively. This is due to the decomposition of magnesium carbonate associated with the decomposition of dolomite (Equation (10)) [36,37,38], with a weight loss, according to the TG curve, of 20 wt % for C_A, 9 wt % for C_SN_1, and 7 wt % for C_SN_2. Subsequently, for C_A two other endothermic reactions occur at 845 °C and 860 °C, respectively, while in C_SN_1 and C_SN_2 a single endothermic reaction is observed at 860 °C, which is due to the decomposition of calcium carbonate, in this case (Equation (9)) [39,40,41,42,43,44], with a weight loss of 28 wt % for C_A, 35 wt % for C_SN_1 and 37 wt % for C_SN_2.

CaCO_3_ (s) → CaO (s) + CO_2_ (g) (9)

CaMg(CO_3_)_2_ (s) → MgO (s) + Ca (s) + 2CO_2_ (g) (10)

Comparing the DSC test curves of the C_A limestone with those of the C_SN_1 and C_SN_2 limestones, the first endothermic peak produced by the decomposition of the carbonate ions associated with magnesium appears in the range of 775–803 °C; that is, the C_A has a higher thermal reaction enthalpy. This reflects an agreement with the previous results and also the higher content of magnesium oxides in its composition. According to the differential scanning calorimetry (DSC) analysis of C_T, the endothermic reaction takes place at about 860 °C [41,42,43,44].

The DSC-TG analyses of clays A_A (Figure 4e), A_N (Figure 4f), and A_R (Figure 4g) show an endothermic peak at temperatures between 20 °C and 200 °C, demonstrating the removal of weakly bound water (dehydration), with a weight loss of 1.7 wt % for A_A, 1.3 wt % for A_N, and 1.7 wt % for A_R. The intensity is slightly higher in the A_A curve, which is possibly due to the presence of montmorillonite [45], as corroborated by XRD analysis. In the temperature range between 200 °C and 600 °C, a weight loss attributed to the removal of organic matter and the dehydroxylation of silicates is observed, as indicated by the exothermic band centered at approximately 380 °C and the endothermic band centered at approximately 550 °C, respectively [46,47]. These weight losses correspond to 1.9 wt % for A_A, 2.4 wt % for A_N, and 3.0 wt % for A_R. The largest weight loss occurs between 600 °C and 800 °C, this being due to the decomposition of carbonates, as indicated by the endothermic peak centered at approximately 730–780 °C, with losses of 9.6 wt % for A_A, 8.7 wt % for A_N, and 5.0 wt % for A_R. At temperatures between 800 °C and 1100 °C, an exothermic band associated with the crystallization of the high-temperature phases is observed, with weight losses of 0.3 wt % for A_A, 2.2 wt % for A_N, and 0.3 wt % for A_R. In this temperature zone, specifically at 900 °C and in A_A, there is a small endothermic effect associated with the conversion of low-temperature albite to its high-temperature phases, although these effects may overlap with those of calcium carbonate decomposition [48]. At temperatures above 1100 °C, no weight loss is observed, with a more pronounced endotherm in the DSC curve in A_A, which could possibly indicate the formation of a liquid phase [6].

In the DSC curve of the sample of gypsum (Y) (Figure 4h), different endothermic peaks and an exothermic peak are observed. Firstly, up to about 110 °C, an endothermic effect occurs, which is due to the loss of moisture in the material, resulting in a weight loss of 1 wt %. Secondly, there are two overlapping endothermic effects corresponding to the dehydration of the gypsum into hemihydrate and anhydrite III. The first of these, produced at 150 °C, corresponds to the loss of 1½ H_2_O to form the calcium sulfate hemihydrate phase (Equation (11)) [49,50], while the second, produced at 190 °C, corresponds to the loss of ½ H_2_O to form the β-anhydrite III or soluble anhydrite phases (Equation (12)) [49,50,51]. These two reactions result in a weight loss of 17.5 wt %.

CaSO_4_·2H_2_O → CaSO_4_·1⁄2H_2_O + 3⁄2H_2_O (11)

CaSO_4_·1⁄2H_2_O → CaSO_4_ (III) + 1⁄2H_2_O (12)

An exothermic reaction associated with the transformation of anhydrite III (soluble) to anhydrite II (insoluble) occurs at 380 °C (Equation (13)) [49,50]. The weight loss between the temperatures of 190 °C and 380 °C is 2 wt %.

CaSO_4_ (soluble) → CaSO_4_ (insoluble) (13)

The next endothermic reaction takes place at 1220 °C and corresponds to the change from anhydrite II to anhydrite I. From this point on, the decomposition of calcium sulfate takes place (Equation (14)) [50], with a weight loss of 3 wt %.

CaSO_4_ (I) → CaO + SO_2_ + 1⁄2O_2_ (14)

In the rather complex thermogravimetric curve of the CHM waste (Figure 4i), two important inflections can be observed, corresponding first to weight loss due to the decomposition of the carbonates (4 wt %) up to 750 °C and a second weight loss of 1.5 wt % between 1100 °C and 1200 °C, due to the elimination of the last traces of structural water and the formation of the amorphous or glassy phase, and also due to the consumption produced by the continuous reactions, such as the formation of anorthite and mullite [3Al_2_O_3_·2SiO_2_]. Other compounds, such as illite and gehlenite [Ca_2_Al_2_SiO_7_], decompose at temperatures above 1150 °C. According to the DSC analysis, a first thermal dehydration process produced by heating can be observed, as indicated by the endothermic reaction at a temperature below 100 °C that is associated with the release of the absorbed water. The second endothermic reaction at 575 °C is due to the polymorphic phase-change of the silica, which generates strong dimensional variations in its structure (Equation (15)) [48].

quartz (α) → (β) quartz(15)

The third endothermic reaction at 731 °C is due to the decomposition of calcite (Equation (9)). Around 1100 °C, an exothermic reaction can be observed, indicating the formation of crystalline phases. At temperatures above 1150 °C, no weight loss is observed, but the DSC curve shows three endothermic peaks, which may indicate the formation of the liquid phase [6].

The TG curve of the MRM waste (Figure 4j) shows a main inflection due to the decomposition of carbonates (Equation (9)) with a weight loss of 44 wt %, in agreement with the results for the loss on ignition (Table 3). According to the DSC analysis, the presence of calcite is deduced through the endothermic reaction that takes place around 866 °C [6].

In the TG curve of the PVL waste (Figure 4k), a total weight loss of 17.2 wt % can be observed, up to a temperature of 1390 °C. According to the DSC curve, an endothermic reaction occurs at 290 °C that is associated with the dehydroxylation of Al(OH)_3_ (Equation (16)) [49], which generates a weight loss of 8.5 wt % [52].

2Al(OH)_3_ → Al_2_O_3_ + 3H_2_O (16)

The sequence of the thermal transformation of alumina can be defined as obtaining γ-Al_2_O_3_ at 500 °C, remaining as a single phase up to 600 °C. Subsequently, at 850 °C, the δ-Al_2_O_3_ phase is identified, which coexists with γ-Al_2_O_3_. At 1000 °C, θ-Al_2_O_3_ is obtained, with the coexistence of the previous phases γ-Al_2_O_3_ and δ-Al_2_O_3_. Subsequently, an exothermic reaction can be seen at about 1150 °C, which is due to the transformation into α-Al_2_O_3_ (corundum). The transformation of metastable alumina into corundum takes place in a temperature range of 1062–1204 °C when the treatment is carried out in an air atmosphere, and between 1090 and 1181 °C when an inert atmosphere (N_2_) is used [53,54].

#### 3.1.4. Scanning Electron Microscopy and Energy-Dispersive X-ray Spectroscopy Analysis (SEM/EDS)

In general, and due to the grinding process to which the materials have previously been subjected, it can be observed via SEM that the particle size obtained in all the materials is less than 100 μm, with grains of angular appearance appearing in many cases due to the grinding process.

According to the EDS analysis carried out on C_A, the composition is dominated by Ca, Mg, and O. There are also other minority elements, such as Al, Si, and Fe. For the limestone C_SN_1, there are zones of different compositions, with Ca, Mg, and O predominating. In general, the presence of Al, Si, and Fe can also be observed, although in a minimum proportion in all the zones. The EDS analysis of sample C_SN_2 indicated that the composition is very similar to that of sample C_SN_1; it should be noted that both samples come from the same raw material. The only difference in both samples is the granulometry prior to the grinding process. The main difference is the appearance of K in one of the areas studied, although in a very small proportion (0.17 wt %). In the sample C_T that is analyzed, the particle size ratio is different from that studied for C_A, C_SN_1, and C_SN_2. A greater number of large particles are observed, although always smaller than 100 μm. This may be due to the greater hardness of the grains in C_T compared to the other three samples since the grinding time in all these samples is the same. In addition to this, it is observed that the larger grains do not have more defined edges or surfaces, which may be due to difficulties regarding the fragmentation of the grains in the grinding process and the need to induce a greater number of impacts to reduce the size, which causes the faces to collide a greater number of times and become more rounded. The smaller particles have better-defined smooth faces. In terms of the EDS analysis, the studied zones can be grouped into three differentiated zones and present a higher Ca and O composition, with a higher wt % of Ca in C_A compared to the other three studied limestones. Different percentages of Mg are also observed, although in a much lower proportion than in the other limestones, coinciding with the XRF results (Table 2), Al, Si, K, and Fe.

In the morphological study of A_A and A_N, very similar particle sizes are observed, with grains of heterogeneous size and different shapes (both rounded and angular). In A_R, the overall particle shape is smaller, being more homogeneous and rounded in most of the grains. According to the EDS analysis, in general, in A_A, A_N, and A_R, the elements with a higher wt % are Si, Al, and O, as expected in the case of clays and coinciding with the XRF analysis (Table 2). In the case of A_A, zones of differentiated composition can be identified, with different percentages of K, Ca, Fe, and, to a lesser extent, Na, Mg, Ti, and Cu. In the case of A_N, zones of differentiated composition can be identified, with different percentages of mainly K and Fe, and Na, Mg, S, Ca, and Ti in relative proportions. In the case of S, for the three clays studied, this element is only identified in the A_N in values lower than 0.5 wt %. For the analysis of A_R, it is possible to identify areas with different compositions; Fe is the element with the highest percentage, in addition to Si, Al, and O, with a higher composition than in the case of A_A and A_N. In addition, the presence of Na, Mg, K, Ca, and Ti can be observed in smaller proportions.

In the case of Y, the particles have a very heterogeneous morphology, with grains smaller than 10 μm, agglomerates, and larger particles that are still smaller than 100 μm. Some particles have an elongated shape, although most of them are rounded. The results of the EDS microanalysis show two zones of differentiated composition. In both zones, the predominant elements are S, Ca, and O, which is in agreement with the XRD and XRF (Table 3) analyses mentioned earlier.

**Figure 5:**
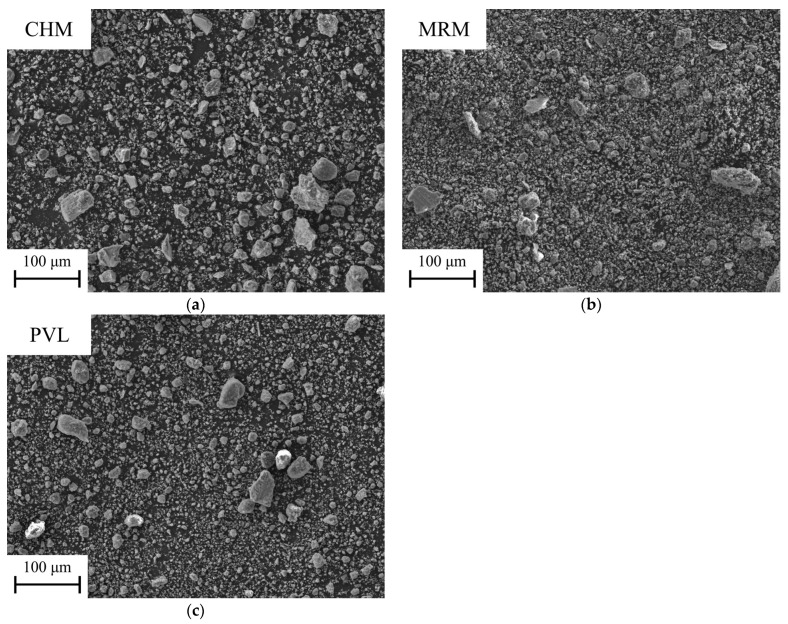
Selected SEM micrographs (×500) of: (**a**) CHM powder; (**b**) MRM powder; (**c**) PVL powder.

In terms of industrial waste, Figure 5a shows a selected SEM micrograph at a 500× magnification of the CHM sample, in which particles of different sizes can be observed, the smaller ones being more numerous and all being smaller than 100 μm. The morphology of the particles is varied, some being particularly rounded and others more angular. The EDS microanalysis shows 5 zones with different compositions (Table 4), although the major elements are Si, Al, and O, followed by K, Ca, Fe, and, to a lesser extent, Na, Mg, P, S, Ti, and Cu. The distribution of the elements in the microanalysis of this sample resembles the composition in the EDS analysis that was conducted on the clays.

In the case of the MRM waste (Figure 5b), the particles are much smaller in size, with a more powdery appearance. In this case, most of the particles are smaller than 50 μm. Agglomerates of the particles and some larger fragments can be observed. The EDS analysis (Table 5) shows three zones with different compositions, in which the elements with the highest wt % are Ca and O, although the differences lie in the wt % of minor elements, such as Mg, S, and Cu, which are always in quantities lower than 0.50 wt %. This composition agrees with the XRD (Figure 3) and XRF (Table 3) results that correspond to calcium carbonate.

Figure 5c shows a selected SEM micrograph of the PVL. Heterogeneous particle sizes are observed, although most of them do not exceed 50 μm. A larger amount of carbon tape (black background) without adhered particles can be observed, which may be due to the shape of the particles, with defined edges, which may hinder adhesion and permanence on this tape. The EDS microanalysis (Table 6) shows 5 point zones with variable composition, although the major elements in all of them are Al and O, which finding is in agreement with the XRF analysis (Table 3). Different compositions can also be observed, depending on the zone, of Mg, Si, and K, as well as F, Na, S, Cl, Ca, Ti, Fe, Cu, and Ba, to a lesser extent,. This composition agrees with the phases identified in the XRD test (Figure 3).

#### 3.1.5. Attenuated Total Reflectance Fourier Transform Infrared Spectroscopy (ATR-FTIR)

**Figure 6:**
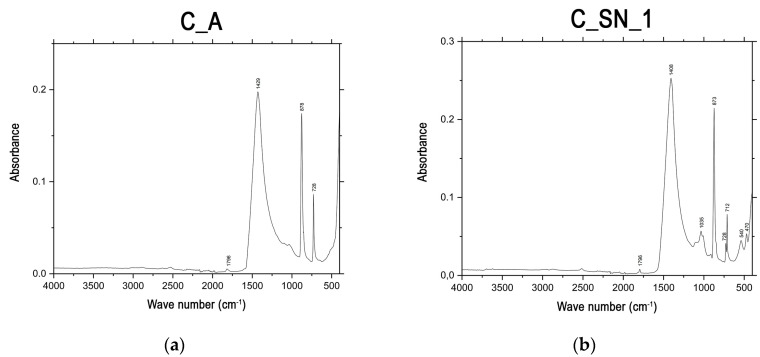

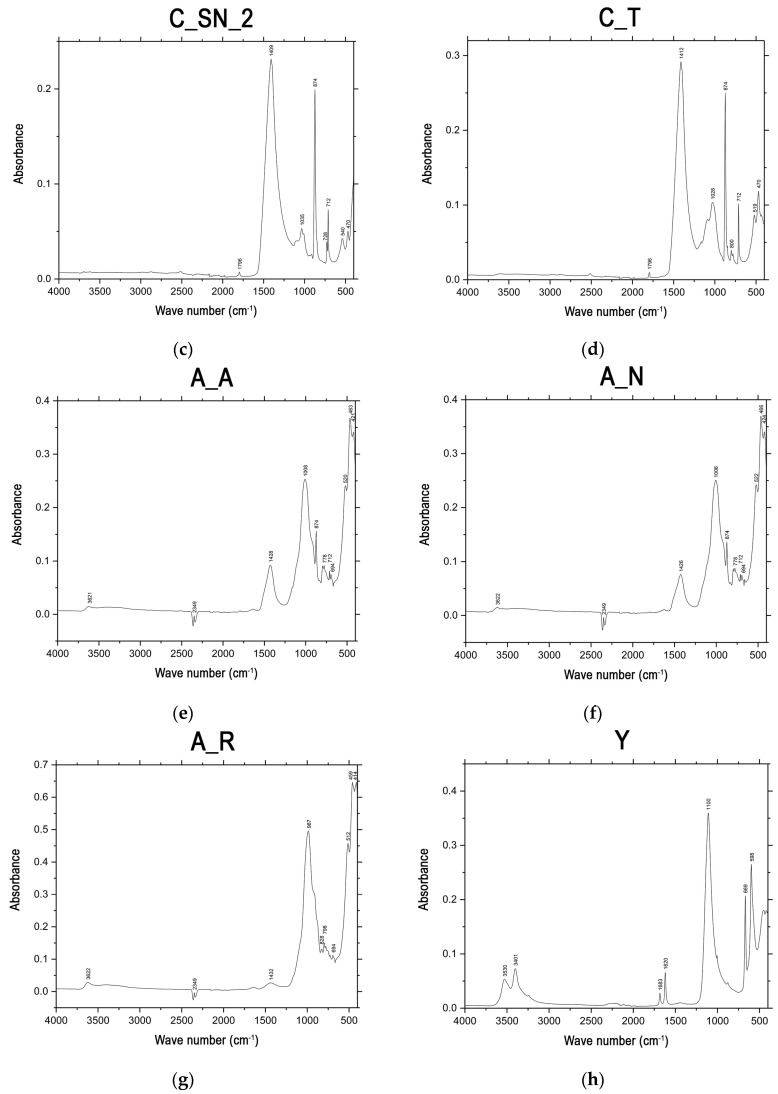

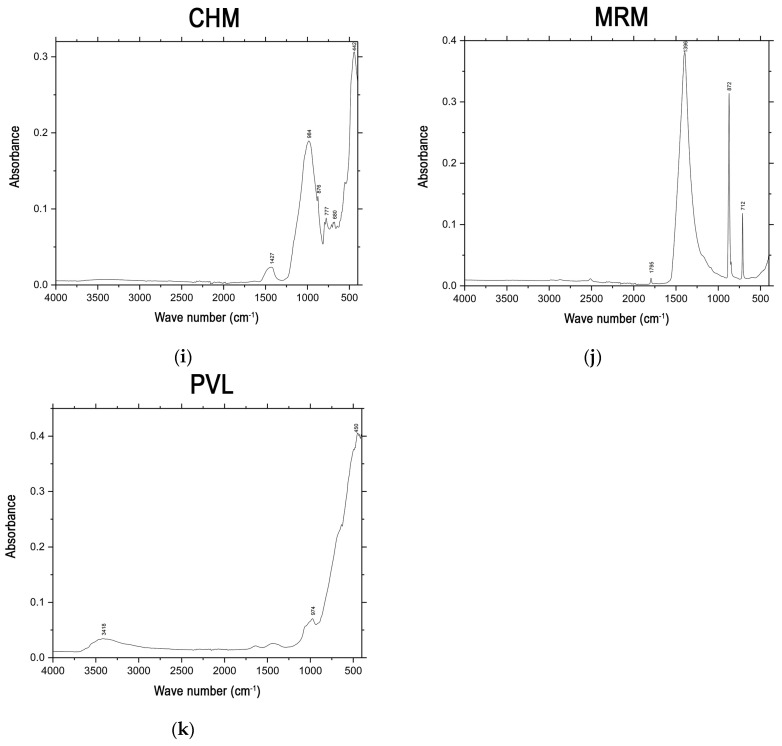
Infrared spectra (ATR-FTIR) of: (**a**) C_A; (**b**) C_SN_1; (**c**) C_SN_2; (**d**) C_T; (**e**) A_A; (**f**) A_N; (**g**) A_R; (**h**) Y; (**i**) CHM; (**j**) MRM; (**k**) PVL.

According to the XRD patterns, C_A, C_SN_1, and C_SN_2 are mineralogically composed of calcite and dolomite. In the case of the C_T limestone, the mineralogical composition is calcite and quartz. Furthermore, according to the results of the XRF analysis, it can be seen that the C_A has a high MgO content, being lower in the case of the C_SN_1 and C_SN_2 limestones, and much lower in the C_T limestone. According to the absorption spectra, C_A (Figure 6a) shows absorption bands corresponding to low-Mg calcite, specifically those centered at 1796 cm^−1^ and 878 cm^−1^, as well as a band centered at 1429 cm^−1^ corresponding to high-Mg calcite. In the absorption spectrum of dolomite [CaMg(CO_3_)_2_], where the structure of the flat CO_3_^2−^ ions is intercalated with the Ca^2+^ and Mg^2+^ cations, the absorption band is close to 729 cm^−1^, and a band centered at 728 cm^−1^ can be observed in the C_A band [55,56,57]. For C_SN_1 (Figure 6b) and C_SN_2 (Figure 6c), absorption bands are shown corresponding to low-Mg calcite, namely, those centered at 1796 cm^−1^, 1408 cm^−1^ and 1409 cm^−1^, 873 cm^−1^ and 874 cm^−1^ and 712 cm^−1^, a band centered at 728 cm^−1^ corresponding to dolomite, two bands centered at 1035 cm^−1^ and 540 cm^−1^ matching the feldspars, and a band centered at 470 cm^−1^ corresponding to the angular deformation of the Si-O-Si bond [55,56,57,58,59,60]. It can be seen that the band centered at 728 cm^−1^ has a higher intensity in the spectrum of C_A compared to the spectra of C_SN_1 and C_SN_2, which is consistent with the higher proportion of dolomite in its composition. This is due to the higher concentration of MgO since, according to the results of the XRF analysis (Table 2), these concentrations vary from 19.12 wt % to 4.59 and 4.63 wt %, respectively. For the C_T sample (Figure 6d), absorption bands corresponding to low-Mg calcite are shown, namely, those centered at 1796 cm^−1^, 1412 cm^−1^, 874 cm^−1^, and 712 cm^−1^, a band centered at 1028 cm^−1^ corresponding to feldspar, and three bands centered at 800 cm^−1^, 519 cm^−1^, and 470 cm^−1^, matching the angular deformation of Si-O-Si. In the case of the band centered at 800 cm^−1^, it can be seen that it is accompanied by another band of lower intensity at approximately 780 cm^−1^. This double band is characteristic of the symmetric Si-O stretching of quartz [55,56,57,58,59,60,61,62].

The study of clays via ATR-FTIR indicates two absorption frequency zones: the OH vibration zone (3200–3700 cm^−1^) and the lattice vibration zone (1100–400 cm^−1^). In general, for the spectra of the three types of clays studied, A_A (Figure 6e), A_N (Figure 6f), and A_R (Figure 6g), the characteristic bands of the silicates are observed, corroborating the presence of O-bonded Si and O-H groups, as evidenced by the Si-O stretching bands at 1200–950 cm^−1^. The doublet bands at approximately 700–800 cm^−1^ reveal the characteristic signatures of the Si-O bond stretching, while the bands at 600–800 cm^−1^ are characteristic of the Si-O bond-bending mode [62]. In the case of the three clays, an absorption band centered at 3622–3621 cm^−1^, corresponding to the stretching band of the structural O-H groups, can be observed [61]. The absorption band centered at 2349 cm^−1^ can be attributed to atmospheric CO_2_ and can also be observed in all three clays [63]. The absorption band centered between 1008 and 987 cm^−1^ represents the in-plane stretching of Si-O in the Si-O-Si groups of the tetrahedral sheet [61]. On the other hand, three absorption bands can be distinguished that correspond to the three different modes of vibration of the C=O bond contained in the carbonate ion [64]. These absorption bands, corresponding to low-Mg calcite, are centered between 1432 and 1426 cm^−1^, due to the asymmetric stretching of the CO_3_^2−^ band, the asymmetric bending mode of the CO_3_^2−^ band at 874 cm^−1^, and the symmetric mode of CO_3_^2−^ centered at 712 cm^−1^, although these last two absorption bands are only found in the A_A and A_N spectra. The presence of quartz is associated with the doublet at 800 cm^−1^ and 778 cm^−1^, respectively, due to the Si-O symmetric stretching mode, as well as the band at 694 cm^−1^ corresponding to Si-O-Al vibrations, and also the bands centered at 522–512 cm^−1^ and 466–459 cm^−1^, which are due to Si-O-Al (octahedral) bending and Si-O-Si angular deformation vibrations, respectively. Finally, the band located at 424–414 cm^−1^ is due to the bending modes of O-Ca-O [55,56,57,58,59,60,61,62,63,65].

In the case of A_N, an absorption band centered at 3621 cm^−1^ is observed, corresponding to the stretching band of the structural O-H groups [61]. Regarding the absorption band centered at 2349 cm^−1^, this can be attributed to atmospheric CO_2_ [63]. The absorption band centered at 1008 cm^−1^ represents the in-plane stretching of Si-O in the Si-O-Si groups of the tetrahedral sheet [61]. The absorption bands corresponding to low-Mg calcite are centered at 1428 cm^−1^ due to the asymmetric stretching of the CO_3_^2−^ band, the asymmetric bending mode of the CO_3_^2−^ band at 874 cm^−1^ and the symmetric mode of CO_3_^2−^ situated at 712 cm^−1^. For quartz, the doublet at 800 cm^−1^ and 778 cm^−1^ can be observed, due to the symmetric Si-O stretching, as well as the band at 694 cm^−1^ that corresponds to the Si-O-Al vibrations and the bands centered at 520 cm^−1^ and 463 cm^−1^, which are due to Si-O-Al (octahedral) bending and the Si-O-Si angular deformation vibrations modes, respectively. The band identified at 421 cm^−1^ is due to the O-Ca-O bending mode. In the case of A_R, an absorption band at 828 cm^−1^ is observed, which can be attributed to the OH deformation of the octahedral layers (Fe^3+^-Fe^3+^-OH) [55,56,57,58,59,60,61,62,63,65].

In the ATR-FTIR spectrum of the gypsum (Y) (Figure 6h), the presence of water can be detected and characterized by bands in the spectral region near 3500 cm^−1^ and 1600 cm^−1^. In this case, a doublet of absorption bands is observed at 3530 cm^−1^ and 3401 cm^−1^, the first one being due to the stretching vibrations of the hydroxyl groups, and the second one being due to the ν_1_ vibrational modes of the symmetric stretching of water [61,66]. In the frequency ranging between 2000 and 1000 cm^−1^ appears in two bands at 1683 cm^−1^ and 1620 cm^−1^, which are assigned to the bending vibrational modes of the O-H bond [66,67]. The presence of two bands indicates that there are two different types of crystallographic water. One type of water is bound to the sulfate ions by hydrogen bonding; the corresponding band has a lower frequency (also due to hydrogen bonding) and high intensity. The other is directly related to calcium ions [66]. There is also a strong band at 1100 cm^−1^, due to the antisymmetric stretching vibration (ν_3_) of the SO_4_ tetrahedra. In addition, there is a very weak band at about 1003 cm^−1^, which represents the symmetric stretching vibration mode (ν_1_) of the SO_4_ tetrahedra. Finally, the spectrum exhibits two antisymmetric bending vibration bands at 669 cm^−1^ and 598 cm^−1^ [66,68].

For the CHM sample (Figure 6i), an absorption band centered at 984 cm^−1^ can be observed, which represents the asymmetric Si-O stretching of the main tetrahedral silicate vibrations [61,69,70]. The absorption band centered at 1427 cm^−1^ is due to the asymmetric stretching (ν_3_) of CO_3_^2−^, while the band centered at 876 cm^−1^ is due to the asymmetric bending mode (ν_2_) of CO_3_^2−^. A doublet at 800 cm^−1^ and 777 cm^−1^ can be observed, due to the symmetric stretching of the Si-O bond, as well as the band at 680 cm^−1^ corresponding to the Si-O-Al vibrations. The absorption band centered at 442 cm^−1^ corresponds to the vibration produced by the angular deformation of Si-O-Si [55,56,57,58,59,60,61,62,63,65].

The analysis of the MRM (Figure 6j) shows absorption bands corresponding to calcium carbonate, as indicated by the XRD test, specifically located at 1795 cm^−1^ (ν_1_ + ν_4_), 1396 cm^−1^ (ν_3_), 872 cm^−1^ (ν_2_) and 712 cm^−1^ (ν_4_) [67,71,72,73]. As indicated above in the analysis of the limestones, the free CO_3_^2−^ ion is flat and causes four intramolecular vibrations, namely, ν_1_ (symmetrical stretching), ν_2_ (bending beyond the plane), ν_3_ (symmetrical stretching), and ν_4_ (bending in the plane) [55].

For the PVL sample (Figure 6k), a spectrum with an absorption band centered at 3418 cm^−1^ is observed, due to the stress mode of the O-H bonds. This band indicates the presence of moisture in the analyzed sample. The band centered at 974 cm^−1^ is due to the α-Al_2_O_3_ phase, while the absorption band centered at 450 cm^−1^ is associated with the stretching mode of Al-O in the octahedral structure [74].

### 3.2. Crude Blend Design

**Figure 7:**
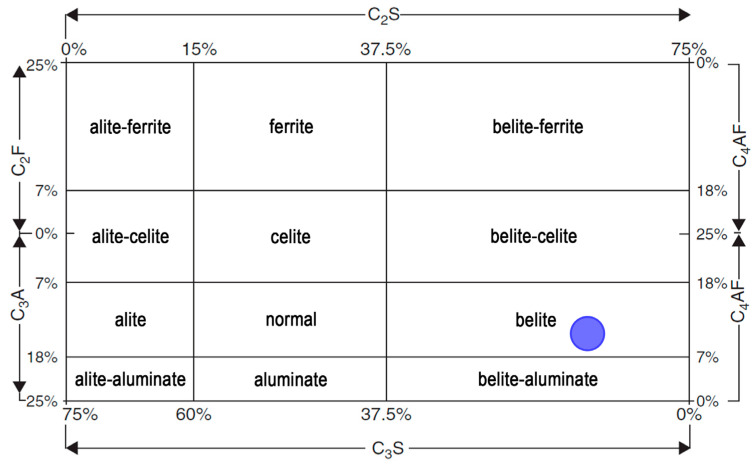
Classification of cement types according to the composition of their majority phases. Blue area: target cement type. (Adapted from reference [77]).

Taking into account all the previous results and the compatibility in the results, which were obtained using raw materials and industrial wastes, the design of the raw blend for low-energy cement using industrial wastes was studied. As indicated above, the main difference between ordinary Portland cement (OPC) and low-energy cement with a high dicalcium silicate content was the preponderance of the type of silicate seen in the final clinker, with the C_3_S/C_2_S weight ratio in the case of OPC being approximately 60%/20% [75], while in the newly designed clinker crudes, the value of the percentage of C_2_S varies up to 60–65% by weight [6]. According to the percentage of C_2_S proposed, the percentage by weight of the rest of the major components of the clinker (C_3_S, C_3_A, and C_4_AF) will vary, taking into account the limitations set by the current regulations on the need for cement clinker to be made up of at least two-thirds of calcium silicates [76]. Figure 7 classifies the different types of cement, according to the majority of phases calculated in the design, with the target zone shown in blue [77].

In the present study, to achieve desirable C_2_S and C_3_S percentages, the LSF should be between 78% and 83% [31,32,78] and, more specifically, within the range of 78–80%, this being appropriate for belite cement [30], in order to obtain high-quality hydraulic cements. Furthermore, it should be taken into account that the mass ratio (CaO)/(SiO_2_) should not be less than 2.0 [76].

Table 7 presents the crude blend formulations designed using the calculation and with a Microsoft Excel^®^ spreadsheet, as described in Section 2.3. The design of three crude blends using only raw materials (WST_0_A, WST_0_SN, and WST_0_T) can be seen, but each of them uses a different limestone (C_A, C_SN, and C_T). Due to the common characteristics of the C_SN_1 and C_SN_2 limestones, analyzed above, which come from the same mining site, the designed blend, WST_0_SN, contains only the limestone C_SN_1. In addition, a crude blend designed using 50 wt % of raw materials (C_T, A_A, A_N, and A_R) and 50 wt % of the industrial wastes previously studied (CHM, MRM, and PVL), known as WST_50_T, as well as a crude blend designed exclusively from industrial wastes, called WST_100, can be observed. These two blends are intended to demonstrate the possibility of manufacturing cement from industrial waste, with either a high percentage of 50% or a total substitution of the raw materials.

Table 8 shows the theoretical compositions of the designed raw blends, in terms of the main cement clinker phases (C_2_S, C_3_S, C_3_A, and C_4_AF), the quality indexes (LSF, AM, and SM). and the CaO/SiO_2_ ratio. It is worth taking note of the design limitations for a C_2_S composition of 60–65 wt %, with an LSF of 78–80 wt % and a CaO/SiO_2_ ratio that is higher than 2.0.

Taking into account the results of the characterization of the C_A limestone, it is necessary to highlight a fundamental fact about this raw material that will be decisive in the calculation of the crude blends. In this case, the chemical composition (Table 2) of C_A shows a percentage of 19.12 wt % of MgO. It is important to keep in mind that, to avoid future cement expansivity problems, its MgO percentage cannot exceed 5 wt % [76]. This characteristic of C_A is also corroborated in the thermal analyses that were performed, with an endothermic peak centered at 803 °C due to the decomposition of the carbonates associated with magnesium. Therefore, for the design of the crude blends with C_A, the values associated with MgO and how these influence the calculations in the designed Microsoft Excel^®^ spreadsheet must be taken into account. Another problem associated with the high MgO content of the blends is the theoretical composition of the clinker target phases (Table 8), since the calculation of these phases is performed by means of the modified Bogue Equations taking into account the four main oxides of the crude, namely Al_2_O_3_, CaO, SiO_2_, and Fe_2_O_3_. Thus, a high MgO content can imply a notorious difference between the theoretically calculated phases and the real manufactured phases. As in the limestone C_A, the chemical composition (Table 2) of C_SN_1 indicates a high percentage of MgO, specifically 4.59 wt %. This percentage is lower than the 5 wt % previously indicated as a limit in the composition of cement clinker, although the 4.59 wt % is referred to the chemical composition without calcination. Therefore, it will increase based on the calcined composition. Consequently, as with the crudes designed with C_A, the values associated with MgO, and its influence on the calculations in the Microsoft Excel^®^ spreadsheet that has been designed, must be taken into account for the crudes designed with C_SN_1.

The results of the chemical composition of the manufactured crudes and the loss on ignition (LOI), are shown in Table 9. These results are reasonable considering the composition of the main phases of the cement clinker to be obtained. It should be noted that in both the WST_0_A and WST_0_SN blends, the percentage of MgO is 13.45 wt % and 4.60 wt %, respectively. This indicates that in both cases the percentage of MgO, according to the calcined base, will be higher than 5.0 wt %. Therefore, they will not be valid clinkers. In the case of the WST_0_T, WST_50_T and WST_100 crude samples, the results of the MgO limiting factor are ~1 wt %.

**Figure 8:**
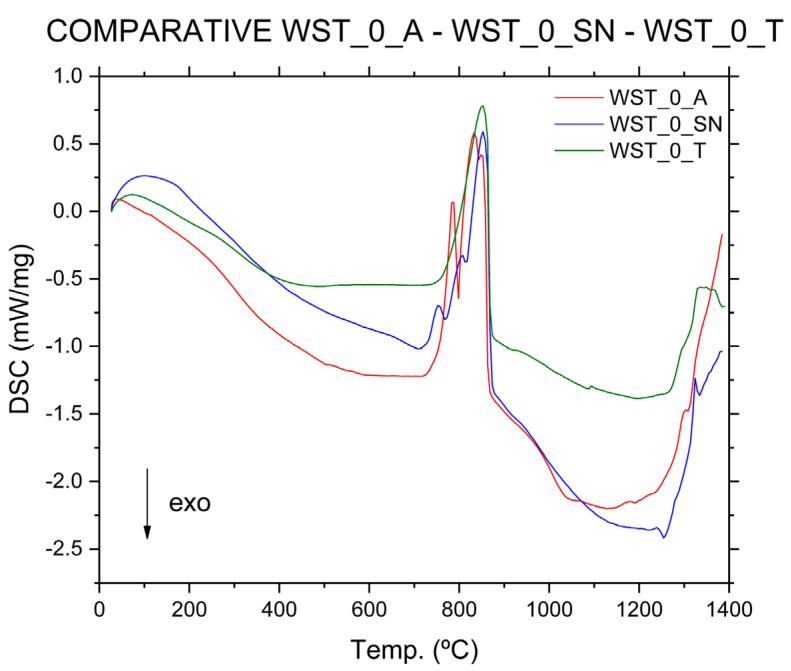
Comparative DSC analysis WST_0_A (red line), WST_0_SN (blue line), and WST_0_T (green line).

According to the DSC analysis performed on the three blends that were manufactured using only raw materials in their composition (Figure 8), it can be seen that in the WST_0_A and WST_0_SN blends, the double decomposition reactions of magnesium and calcium carbonates occur, while in the WST_0_T crude, only an endothermic reaction occurs in the same temperature range for the CaCO_3_ decomposition, due to the low MgO composition of the mixture. This corroborates the data obtained in the XRF analysis for these three blends (Table 7), which allows us to definitively discard the WST_0_A and WST_0_SN crude samples.

**Figure 9:**
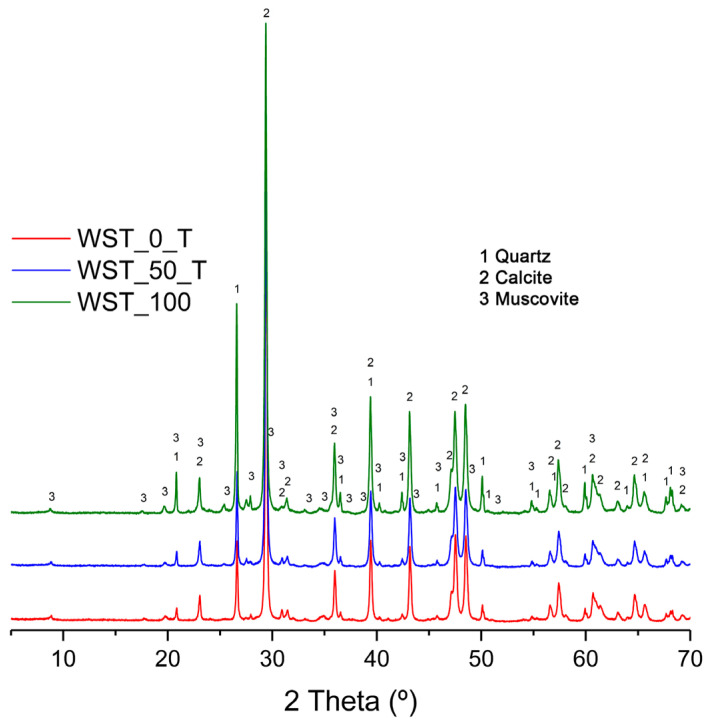
Comparative XRD analysis: WST_0_T (red line), WST_50_T (blue line), and WST_100 (green line).

The mineralogical phases identified by XRD (Figure 9) for the WST_0_T crude blend, the one produced with 50 wt % waste substitution (WST_50_T) and the one produced with 100 wt % waste in its composition (WST_100), are quartz, calcite, and muscovite. It should be noted that in the case of the WST_0_T blend, part of the raw materials used are clays. In the case of the WST_50_T blend and WST_100 sample, clays are replaced by 50 wt % or 100 wt % by CHM, respectively. Thus, in the case of WST_50_T and WST_100 crudes, part of the identified muscovite will be dehydroxylated as it comes from CHM.

It can be observed that the three blends have a similar XRD pattern (Figure 9), regardless of the use or non-use of wastes in their composition and according to the chemical composition results (Table 7). It can be seen how, in these three blends, the presence of dolomite is not identified, due to the low MgO content of the C_T and the CHM and MRM wastes (Table 2 and Table 3). In addition, the phases provided by PVL are not identified in the XRD diffractograms of the WST_50_T and WST_100 blends, since they only represent 0.50 wt % and 1 wt % of the total blend, respectively (Table 7).

Thus, a peak of maximum intensity corresponding to calcite in the 28–30° 2θ zone can be observed for the XRD patterns (Figure 9) of WST_0_T and WST_50_T. In the case of the sample WST_100, two peaks of maximum intensity can be observed in the 25–30° 2θ zone. The first one corresponded to quartz and the second one corresponded to a higher-intensity peak for calcite. It is consistent with the current findings that the peak of higher intensity corresponds to calcite, since it is the main mineralogical phase in the MRM, accounting for 37.50 wt % (WST_50_T) and 68.25 wt % (WST_100) of the total crude blend (Table 7). In general, as can be seen in Figure 9, the identified peaks of quartz and muscovite present a higher intensity in the XRD of the WST_100 crude blend, compared to the crude blends made with C_T. This may be caused by the higher composition of CHM in the WST_100 crude sample versus the sum of the compositions of A_A, A_N, and A_R, and CHM in the crude sample manufactured with C_T. Therefore, a lower composition of MRM in this crude sample is reached, compared to the sum of C_T and MRM in the other two crude samples.

**Figure 10:**
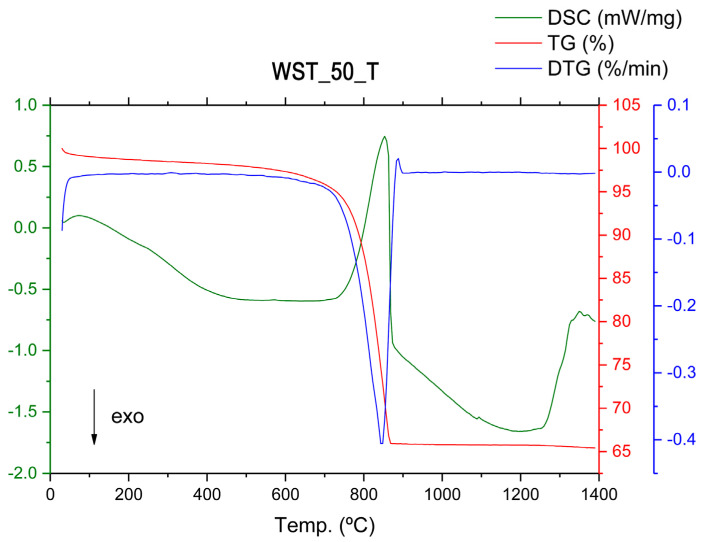
The TG (red line), DTG (blue line), and DSC (green line) analyses of crude-blend WST_50_T.

**Figure 11:**
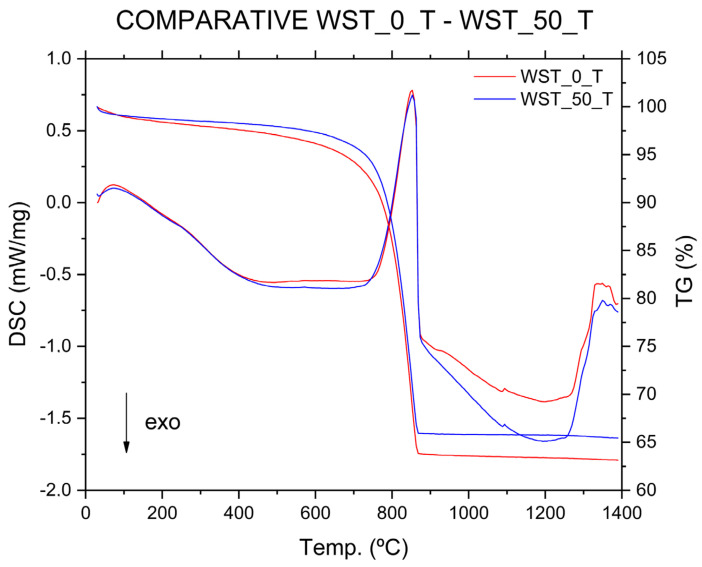
Analyses of WST_0_T (TG (red line), DSC (red line)) vs. WST_50_T (TG (blue line), DSC (blue line)).

Thermal analyses performed on the C_T blends (Figure 10 and Figure 11), composed of quartz, calcite, muscovite, and dehydroxylated muscovite, according to the XRD patterns, show similar results for the two compositions. According to the TG test, there is a total weight loss of up to about 860 °C of 36.5 wt % for the WST_0_T crude blend and 34 wt % for the WST_50_T crude blend. This lower weight loss in the crude blend in which the wastes have been incorporated may be due to the lower ignition loss of CHM compared to clays, which is not compensated for by the higher ignition loss of MRM, compared to C_T (Table 2 and Table 3). Conversely, the reactions that occur in the DSC analysis curves have similarities with the analysis curves of the other crude blends that were studied previously. The initial weight loss that occurs at temperatures between 30 °C and 200 °C is due to the elimination of weakly bound water (dehydration) [6]. The weight losses produced at temperatures ranging from 200 °C to 600 °C, associated with the endothermic band observed in the DSC analysis curves, are explained by taking into account the elimination of organic matter and the dehydroxylation of the hydroxylated silicates that come mainly from the clay components [6]. The weight loss of approximately 29 wt % for the WST_0_T and WST_50_T blends between 600 °C and 860 °C is due to the decomposition of calcium carbonate (calcite) [6,44]. As in the DSC curve of the WST_0_T crude blend, only one endothermic reaction occurs in the same temperature range for the decomposition of calcium carbonate in the WST_50_T crude blend, due to the low MgO composition of the mixture (Figure 11). Between 1050 °C and 1200 °C, different reactions occur, which are due, firstly, to the combination of CaO and SiO_2_, forming the C_2_S phase, along with the combination of CaO with Al_2_O_3_ and Fe_2_O_3_, to form C_4_AF. At temperatures above 1200 °C, reactions occur from the combination of CaO with SiO_2_, to form C_3_S, and from CaO with Al_2_O_3_, to form C_3_A aluminate [6].

**Figure 12:**
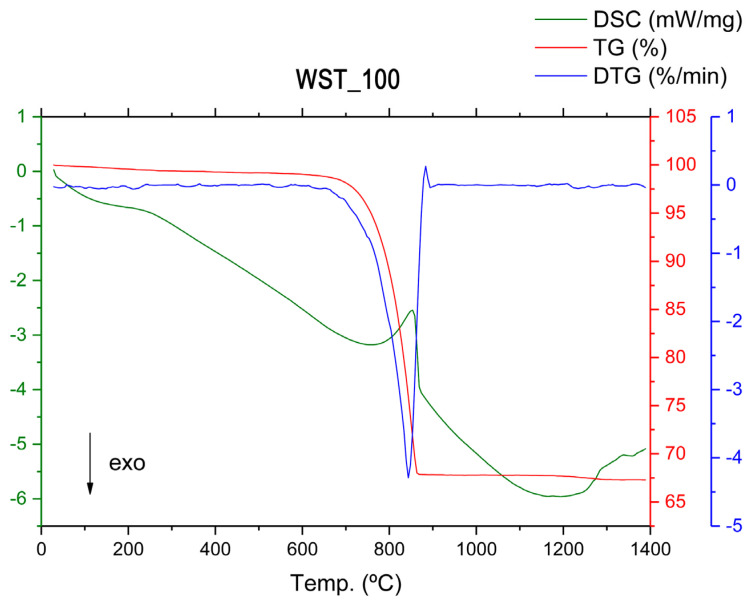
The TG (red line), DTG (blue line), and DSC (green line) analyses of crude-blend WST_100.

According to the thermogravimetric TG curve (Figure 12), in the case of the WST_100 crude blend, there is a total weight loss of up to ~860 °C of 32 wt %. This total weight loss value for the sample is lower than the weight losses indicated in the TG curves for the WST_0_T and WST_50_T blends, which were recorded as 36.5 wt % and 34 wt %, respectively. The lower weight loss, in the WST_100 crude sample, is due to the composition of MRM being lower than that of the sum of C_T and MRM in the other two blends, as well as being due to CHM having a lower LOI compared to clays. Thus, we have a first value that indicates that the CO_2_ emissions of this type of crude blend in the clinkering process, at the same process temperature, will be lower than in the rest of the blends studied.

As in the previous crude blends studied, the initial weight loss that occurs at temperatures between 30 °C and 200 °C is due to the elimination of weakly bound water (dehydration). At temperatures ranging from 200 °C to 600 °C, a weight loss of approximately 5 wt % occurs, due to the elimination of organic matter and the dehydroxylation of hydroxylated silicates, which is associated with the endothermic band shown in the DSC analysis curve (Figure 12). This is followed by a weight loss of approximately 27 wt %, up to 860 °C, which is due to the decomposition of calcium carbonate (calcite). It can be seen that the endothermic effect at 860 °C has a lower intensity in the WST_100 crude sample, compared to the previously analyzed blends. This single effect reinforces the analysis of the low MgO content of the crude sample since a second effect associated with the decomposition of magnesium-associated carbonates does not appear. Between 1050 °C and 1200 °C, as well as above 1200 °C, the same reactions occur as are indicated for the WST_0_T and WST_50_T crude samples.

## 4. Conclusions

The results obtained from the experimental work carried out in this research, as outlined above, focused on the study of crude blends for creating low-energy cement from industrial waste, allowing us to draw the following conclusions.

The main morphological features of the raw materials and industrial wastes have been revealed via SEM studies. It has been deduced that a grinding treatment has been performed in the majority of these materials, resulting in the formation of grains of angular appearance, with a variation in average particle size lower than 100 µm. However, it has been found that polishing-marble sludges are constituted by particles smaller than 50 µm. In the case of PAVAL, heterogeneous particle sizes have been identified, although the 50 µm-diameter measurement is not exceeded. EDS analyses allowed the identification of the main chemical elements that constitute the different powders.

The mineralogical composition of all the samples has been determined by XRD. Calcite and quartz constitute the main mineralogical phases of the limestones, although dolomite has been identified in some samples. Thus, if MgO is present in the chemical composition, it is associated with dolomite. In contrast, the marble residual sludges are constituted by calcite as a single mineralogical phase. The mineralogical composition of the clays, as deduced by XRD, demonstrated the presence of muscovite (illite mica), montmorillonite, and chlorite as clay minerals, and additional silicates, such as feldspars (orthoclase and albite), apart from quartz. The main mineralogical phases identified in the chamotte are feldspars, quartz, calcite, and hematite, with dehydroxylated muscovite and anorthite. The PAVAL sample is constituted of several aluminum hydroxides and oxides, periclase, and quartz.

The application of the ATR-FTIR technique confirmed the mineralogical composition of all the samples studied in this work.

The results of the bulk chemical analysis by XRF have been found to be in good agreement with the EDS analyses of the powders. The chemical composition results have been considered to be of great interest and application in this investigation of the new types of dosages for the manufacture of more sustainable and low-energy cements. It was determined by XRF that the CaO in the limestones ranges between 34.54 and 46.54 wt %, with differences in the MgO content. This is a determining factor in the calculation of the raw material dosages, with implications and the effect of MgO in the expansivity of the produced cements.

The effect of the addition of different industrial wastes for the substitution of raw materials in the production of low-energy cements with high dicalcium silicate content has been studied. Thus, the dosage design has been performed in this investigation, using the Bogue equations and quality indexes (LSF, AM, and SM). The calculations of both the Bogue equations and quality indexes imply the knowledge of the weight percentages of CaO, SiO_2_, Al_2_O_3_, and Fe_2_O_3_, as determined by XRF. In this theoretical design of the different crude samples, a dicalcium silicate ratio of 60–65 wt % and an LSF of 78–83% has been established as a limit value common to all the samples. The calculation basis for the crude blends has been based on calcined materials. Therefore, this has been established as the chemical composition, following this premise.

Thermal analysis by simultaneous TG-DTG-DSC has allowed for checking the mineralogical composition that was deduced by XRD, along with the decomposition and weight loss of calcite and dolomite in the case of the limestone samples and the dehydroxylation of clay minerals in the clay samples, by progressive heating.

By means of the thermal analysis carried out on the crude blends of cement, we have been able to verify the reactions produced in the range of ~700–850 °C as the main difference between blends, due to the decomposition of the carbonates associated with magnesium and calcium. These reactions have a greater or lesser intensity, depending on the composition of the raw materials and the waste used, which allows us to identify the critical compositions for the future characteristics of the cement. Thus, compositions using certain types of limestone with a high MgO content have been discarded. In the case of the thermal analyses, carried out on the crude-blend WST_0_T, using only the raw materials for WST_50_T, which is composed of 50 wt % of raw materials and wastes, and WST_100, which is composed entirely (100 wt %) of industrial wastes, the decomposition reaction of the carbonate associated with the magnesium is not identified. This means that there is less risk of expansivity problems in the manufactured cement.

## 5. Significance and Practical Application of this Investigation

Several recent research efforts have been performed in the utilization of certain industrial wastes for eco-friendly cement production [79,80,81]. For instance, El-Gamal and Selim [79] used ground and granulated blast-furnace slag to produce geopolymer cement at ambient temperature and at about 100% relative humidity. Azad et al. [80] studied the utilization of industrial by-products/waste to manufacture geopolymer cement/concrete. These authors, on the basis of the sustainability indicators, suggested that most of the geopolymers developed using industrial waste leave a positive impact on the environment, economy, and society. More recently, Pinto Ferreira Brasileiro et al. [81] studied self-healing concrete as a way to establish planning for the synthesis and/or characterization of self-healing concretes, reductions in the consumption of cement, and the production of CO_2_. However, in connection with the present investigation, it is interesting that Singh and Subramanian [82] previously explored the production of cement and clinker on a large scale, using post-industrial waste materials from different sources. This research provided the fundamental basis for an environmentally sustainable utilization of these kinds of industrial waste in the production of clinker, thereby making it suitable for use in construction.

In the present work, different types of Spanish and Greek raw materials and industrial wastes (8 raw materials and 3 industrial waste samples) have been identified with the necessary physicochemical characteristics, as well as high generation capacity, to study their possible use as alternative raw materials in the manufacture of low-energy cement. The wastes utilized in this study can be sourced all over the world, as the ceramic industry, the marble extraction and processing industry, and the secondary aluminum industry can be found in all five continents. It should be noted that selected industrial wastes are currently being deposited in landfill sites.

The clinkerization process, as described in the present investigation and in previous precedents [82], is one of the main options for their valorization and their consideration as “technological nutrients”, with researchers working to include them as raw materials within the circularity of the cement industry’s production process. Thus, in this way, the amount of traditional raw materials to be extracted from nature will be reduced, in turn, reducing the volumes to be deposited in landfill sites, with the consequent environmental improvement of the area and a reduction in all the polluting processes associated with mining extraction works. In this sense, wastes are used as raw materials for the production of a more useful material (in the present study, low-energy cement), taking into account the main principle of the circular economy. In fact, a new, 100% waste-based cement clinker blend has been found, using industrial waste as a practical application. This is in line with research concerning news on 100% waste-based materials, such as waste-based mortars and pastes [83].

The next step in this investigation will be the clinkerization process and the production of innovative cements using the waste materials at the laboratory on a pilot scale, as this previous characterization work has revealed a set of promising results.

## Figures and Tables

**Table 1:** Moisture content (wt %) of raw materials, as received in the laboratory.

Raw Materials	Moisture Content (wt %)
C_A	14.5 ± 1.90
C_SN_1	21.7 ± 3.51
C_SN_2	19.3 ± 1.97
C_T	13.1 ± 0.93
A_A	25.3 ± 1.76
A_N	24.7 ± 0.92
A_R	25.4 ± 0.98
CHM	7.1 ± 0.93
MRM	13.3 ± 2.20
PVL	14.6 ± 1.08

**Table 2:** Chemical composition (XRF, wt %) of the raw materials. N.D.: not detected.

	C_A	C_SN_1	C_SN_2	C_T	A_A	A_N	A_R
Al_2_O_3_	0.00	2.44	0.96	1.17	10.53	10.96	19.81
CaO	34.54	41.86	42.77	46.54	17.30	17.41	3.89
SiO_2_	0.00	4.56	2.31	10.51	48.60	44.29	48.69
Fe_2_O_3_	0.26	0.47	0.22	0.86	5.33	5.56	7.71
Na_2_O	0.00	0.17	0.15	0.11	0.40	0.40	0.22
K_2_O	0.01	0.16	0.05	0.29	2.30	2.92	5.47
MgO	19.12	4.59	4.63	0.63	1.86	1.79	3.43
SO_3_	0.02	0.00	0.00	0.00	0.09	8.26	0.05
TiO_2_	0.02	0.05	0.00	0.00	0.64	0.65	0.80
P_2_O_5_	0.00	0.02	0.01	0.00	0.08	0.07	0.10
MnO	0.03	0.00	0.00	0.00	0.03	0.03	0.17
LOI	46.31	43.90	44.20	38.45	12.23	10.70	9.07
Zr (ppm)	6.80	0.00	0.00	0.00	314.20	304.10	136.90
Cl (ppm)	0.06	0.00	0.00	0.00	0.01	0.02	0.02
Cr (ppm)	2.89	0.00	0.00	0.00	0.00	0.00	0.01
Sr (ppm)	N.D.	N.D.	N.D.	N.D.	0.04	0.05	0.02
Ce (ppm)	N.D.	N.D.	N.D.	N.D.	0.00	0.01	0.01
Pb (ppm)	N.D.	N.D.	N.D.	N.D.	0.00	0.00	0.01
Ni (ppm)	N.D.	N.D.	N.D.	N.D.	0.01	0.01	0.01
Cu (ppm)	N.D.	N.D.	N.D.	N.D.	0.01	0.00	0.00
Zn (ppm)	N.D.	N.D.	N.D.	N.D.	0.01	0.01	0.01
Ba (ppm)	N.D.	N.D.	N.D.	N.D.	0.07	0.06	0.07
Rb (ppm)	N.D.	N.D.	N.D.	N.D.	0.01	0.01	0.03

**Table 3:** Chemical composition (XRF, wt %) of the gypsum and the sample wastes.

	Y	CHM	MRM	PVL
Al_2_O_3_	0.18	13.75	0.00	65.40
CaO	24.25	14.75	52.14	1.73
SiO_2_	12.51	51.23	0.00	5.07
Fe_2_O_3_	0.04	6.92	0.06	1.94
Na_2_O	0.00	0.43	0.00	1.24
K_2_O	0.00	3.11	0.00	0.49
MgO	1.81	2.38	0.12	8.28
SO_3_	38.53	3.63	0.20	0.41
TiO_2_	0.00	0.72	0.01	0.67
P_2_O_5_	0.00	0.09	0.00	0.02
MnO	0.00	0.04	0.00	0.18
LOI	22.69	4.00	43.31	12.29
Zr (ppm)	0.00	278.10	5.50	129.70
Cl (ppm)	0.00	0.01	0.01	0.65
Cr (ppm)	0.00	0.01	0.00	0.00
Sr (ppm)	0.00	0.05	0.01	0.04
Pb (ppm)	0.00	0.01	0.00	0.02
Bi (ppm)	0.00	0.00	0.00	0.01
F (ppm)	0.00	0.00	0.00	0.98
Ni (ppm)	0.00	0.01	0.00	0.05
Cu (ppm)	0.00	0.01	0.00	0.45
Zn (ppm)	0.00	0.02	0.00	0.19
Sn (ppm)	0.00	0.00	0.00	0.01
Ba (ppm)	0.00	0.07	0.00	0.20
Rb (ppm)	0.00	0.02	0.00	0.00

**Table 4:** EDS microanalysis (wt %) of the areas marked in Figure 5a (CHM powder).

Element (wt %)	Zone				
	1	2	3	4	5
O	50.69 ± 0.00	39.84 ± 0.00	51.36 ± 0.00	59.38 ± 0.00	61.77 ± 0.00
Na	0.35 ± 0.00	0.18 ± 0.00	0.29 ± 0.00	0.00 ± 0.00	0.00 ± 0.00
Mg	0.99 ± 0.00	1.54 ± 0.00	1.68 ± 0.00	2.21 ± 0.00	0.14 ± 0.00
Al	13.86 ± 0.00	9.00 ± 0.00	9.82 ± 0.00	7.06 ± 0.00	0.51 ± 0.00
Si	24.58 ± 0.00	29.43 ± 0.00	21.95 ± 0.00	13.75 ± 0.00	36.71 ± 0.00
P	0.00 ± 0.00	0.00 ± 0.00	0.00 ± 0.00	0.42 ± 0.00	0.00 ± 0.00
S	0.27 ± 0.00	1.46 ± 0.00	1.49 ± 0.00	0.75 ± 0.00	0.00 ± 0.00
K	6.46 ± 0.00	4.88 ± 0.00	3.66 ± 0.00	1.95 ± 0.00	0.13 ± 0.00
Ca	1.12 ± 0.00	7.03 ± 0.00	6.63 ± 0.00	10.48 ± 0.00	0.35 ± 0.00
Ti	0.00 ± 0.00	0.53 ± 0.00	0.13 ± 0.00	0.00 ± 0.00	0.00 ± 0.00
Fe	1.68 ± 0.00	6.11 ± 0.00	2.69 ± 0.00	4.00 ± 0.00	0.20 ± 0.00
Cu	0.00 ± 0.00	0.00 ± 0.00	0.30 ± 0.00	0.00 ± 0.00	0.19 ± 0.00
Total	100.00 ± 0.00	100.00 ± 0.00	100.00 ± 0.00	100.00 ± 0.00	100.00 ± 0.00

**Table 5:** EDS microanalysis (wt %) of the areas marked in Figure 5b (MRM powder).

Element (wt %)	Zone		
	1	2	3
O	62.06 ± 2.67	61.94 ± 2.67	65.39 ± 0.00
Mg	0.28 ± 0.02	0.33 ± 0.02	0.00 ± 0.00
S	0.00 ± 0.00	0.16 ± 0.00	0.14 ± 0.00
Ca	37.67 ± 2.69	37.58 ± 2.69	34.17 ± 0.00
Cu	0.00 ± 0.00	0.00 ± 0.00	0.30 ± 0.00
Total	100.00 ± 0.00	100.00 ± 0.00	100.00 ± 0.00

**Table 6:** EDS microanalysis (wt %) of the areas marked in Figure 5c (PVL powder).

Element (wt %)	Zone				
	1	2	3	4	5
O	57.76 ± 0.00	29.56 ± 0.00	55.04 ± 0.00	50.41 ± 0.00	48.20 ± 0.00
F	0.00 ± 0.00	0.00 ± 0.00	1.83 ± 0.00	1.20 ± 0.00	1.24 ± 0.00
Na	0.72 ± 0.00	0.79 ± 0.00	1.20 ± 0.00	1.03 ± 0.00	0.55 ± 0.00
Mg	2.46 ± 0.00	10.89 ± 0.00	3.72 ± 0.00	4.64 ± 0.00	2.92 ± 0.00
Al	17.05 ± 0.00	42.68 ± 0.00	29.61 ± 0.00	35.94 ± 0.00	43.35 ± 0.00
Si	12.97 ± 0.00	7.21 ± 0.00	5.89 ± 0.00	2.07 ± 0.00	1.63 ± 0.00
S	0.00 ± 0.00	0.00 ± 0.00	0.21 ± 0.00	0.19 ± 0.00	0.00 ± 0.00
Cl	0.55 ± 0.00	0.98 ± 0.00	0.56 ± 0.00	0.63 ± 0.00	0.41 ± 0.00
K	6.60 ± 0.00	1.78 ± 0.00	0.27 ± 0.00	0.74 ± 0.00	0.39 ± 0.00
Ca	1.89 ± 0.00	1.01 ± 0.00	0.56 ± 0.00	1.37 ± 0.00	0.47 ± 0.00
Ti	0.00 ± 0.00	0.00 ± 0.00	0.16 ± 0.00	0.00 ± 0.00	0.00 ± 0.00
Fe	0.00 ± 0.00	2.63 ± 0.00	0.31 ± 0.00	0.52 ± 0.00	0.45 ± 0.00
Cu	0.00 ± 0.00	1.05 ± 0.00	0.64 ± 0.00	0.49 ± 0.00	0.39 ± 0.00
Ba	0.00 ± 0.00	1.42 ± 0.00	0.00 ± 0.00	0.77 ± 0.00	0.00 ± 0.00
Total	100.00 ± 0.00	100.00 ± 0.00	100.00 ± 0.00	100.00 ± 0.00	100.00 ± 0.00

**Table 7:** Composition (wt %) of the raw materials and wastes of the designed crude blends.

	C_A	C_SN	C_T	A_A	A_N	A_R	CHM	MRM	PVL	Total
WST_0_A	75.85	0.00	0.00	4.17	9.32	10.66	0.00	0.00	0.00	100.00
WST_0_SN	0.00	78.75	0.00	11.50	2.25	7.50	0.00	0.00	0.00	100.00
WST_0_T	0.00	0.00	84.00	5.00	0.75	10.25	0.00	0.00	0.00	100.00
WST_50_T	0.00	0.00	37.50	7.00	0.50	5.00	12.00	37.50	0.50	100.00
WST_100	0.00	0.00	0.00	0.00	0.00	0.00	30.75	68.25	1.00	100.00

**Table 8:** Composition of target phases (wt %), quality indexes (LSF, AM, and SM), and weight ratio (CaO/SiO_2_) of the crude blends designed in this study.

	C_3_S	C_2_S	C_3_A	C_4_AF	Total	LSF	AM	SM	CaO/SiO_2_
WST_0_A	11.32	62.73	14.22	11.73	100.00	78.00	2.03	2.13	2.55
WST_0_SN	8.76	64.14	17.93	9.17	100.00	78.24	2.88	2.11	2.58
WST_0_T	19.00	61.71	10.43	8.86	100.00	78.03	1.99	3.05	2.44
WST_50_T	16.04	61.94	12.44	9.58	100.00	78.18	2.13	2.62	2.49
WST_100	11.72	63.06	14.66	10.57	100.00	78.04	2.23	2.24	2.54

**Table 9:** Chemical composition (XRF, wt %) of the designed crude blends.

	WST_0_A	WST_0_SN	WST_0_T	WST_50_T	WST_100
Al_2_O_3_	4.64	5.14	4.67	4.22	4.46
CaO	26.03	37.35	41.17	44.16	46.49
SiO_2_	13.55	12.81	15.89	14.27	12.61
Fe_2_O_3_	1.65	1.69	1.82	1.80	1.96
Na_2_O	0.14	0.18	0.09	0.13	0.20
K_2_O	0.95	0.90	1.11	1.00	0.97
MgO	13.45	4.60	1.10	0.95	0.94
SO_3_	0.26	0.13	0.06	0.40	0.72
TiO_2_	0.19	0.25	0.23	0.22	0.29
P_2_O_5_	0.05	0.04	0.08	0.05	0.03
MnO	0.04	0.02	0.06	0.04	0.02
LOI	38.80	36.85	33.62	32.67	31.24

## Data Availability

Not applicable.

## Appendix A

**Table A1:** List of notations/abbreviations ordered according to their occurrence in the text.

	Notation/Abbreviation
Greenhouse gas emissions	GHG
Carbon dioxide	CO_2_
Conference of the Parties to the United Nations Framework Convention on Climate Change	COP
Conference of the Parties serving as the meeting of the Parties to the Kyoto Protocol	COP-MOP
European Union	EU
Megatonne	Mt
Percentage	%
Calcium carbonate, calcite	CaCO_3_
Calcium oxide	CaO
Kilogram	kg
Kilowatt hours	kWh
Ordinary Portland cement	OPC
Silicon dioxide, quartz	SiO_2_
Aluminum oxide	Al_2_O_3_
Ferric Oxide, hematite	Fe_2_O_3_
Alite or tricalcium silicate	C_3_S
Belite or dicalcium silicate	C_2_S
Tricalcium aluminate	C_3_A
Tetracalcium alumino-ferrite	C_4_AF
Belitic Portland cement	BPC
Celsius	°C
Kilojoule	kJ
Belite polymorphs	α, α’H, α’L, β, and γ
Hour	h
Percentage by weight	Wt %
Magnesium oxide, periclase	MgO
Limestone ANAYA	C_A
Association Limited	S.L.
Limestone SANTO NICASIO 1	C_SN_1
Limestone SANTO NICASIO 2	C_SN_2
Limestone TITAN	C_T
Yellow clay	A_A
Black clay	A_N
Red clay	A_R
Chamotte	CHM
Société Anonyme	SA
Cutting and polishing marble sludges	MRM
Millimeter	mm
Secondary aluminium production of salt slag	PVL
Gypsum	Y
Initial material sample weight	m_i_
Final material sample weight	m_f_
Micrometer	μm
X-ray fluorescence	XRF
United Kingdom	UK
X-ray diffraction	XRD
Thermogravimetric analysis	TG
Derivative thermogravimetric analysis	DTG
Differential scanning calorimetry	DSC
Scanning electron microscopy	SEM
Energy dispersive X-ray spectroscopy analysis	EDS
Attenuated total reflectance Fourier transform infrared spectroscopy	ATR-FTIR
United States of America	USA
Lime Saturation Factor	LSF
Aluminum Modulus	AM
Silica Modulus	SM
Loss on ignition	LOI
Not detected	N.D.
Sodium Oxide	Na_2_O
Potassium oxide	K_2_O
Sulfur trioxide	SO_3_
Titanium dioxide, rutile	TiO_2_
Phosphorus pentoxide	P_2_O_5_
Manganese oxide	MnO
Zirconium	Zr
Parts per million	ppm
Chlorine	Cl
Chromium	Cr
Strontium	Sr
Cerium	Ce
Lead	Pb
Nickel	Ni
Copper	Cu
Zinc	Zn
Barium	Ba
Rubidium	Rb
Bismuth	Bi
Fluorine	F
Tin	Sn
Dolomite	CaMg(CO_3_)_2_
Muscovite (mica, illite)	KAl_2_(AlSi3O_10_)(OH)_2_
Orthoclase	KAlSi_3_O_8_
Albite	NaAlSi_3_O_8_
Montmorillonite	MgOAl_2_O_35_SiO_2_xH_2_O
Bassanite or gypsum hemihydrate	CaSO_4_.1/2H_2_O
Chlorite	(Al,Fe,Mg)_4–6_(Al,Si,Fe)_4_O_10_(OH,O)_8_
Calcium sulphate dihydrate	CaSO_4_-2H_2_O
Anhydrite	CaSO_4_
Anorthite	CaAl_2_Si_2_O_8_
Corundum	α-Al_2_O_3_
Bayerite	γ-Al(OH)_3_
Nordstrandite	Al(OH)_3_
Diaoyudaoite	NaAl_11_O_17_
Fluorite	CaF_2_
Halite	NaCl
Arbitrary units	a.u.
Solid	s
Gas	g
Mullite	3Al_2_O_3_·2SiO_2_
Gehlenite	Ca_2_Al_2_SiO_7_
Molecular nitrogen	N_2_
Reciprocal centimeter or wave number	cm^−1^
Symmetrical stretching	ν_1_
Bending beyond the plane	ν_2_
Symmetrical stretching	ν_3_
Bending in the plane	ν_4_
Blend containing only raw materials and C_A	WST_0_A
Blend containing only raw materials and C_SN	WST_0_SN
Blend containing only raw materials and C_T	WST_0_T
Blend containing 50 wt % of raw materials, 50 wt % of wastes and C_T	WST_50_T
Blend containing only wastes	WST_100
